# Metabolic Comparison and Molecular Networking of Antimicrobials in *Streptomyces* Species

**DOI:** 10.3390/ijms25084193

**Published:** 2024-04-10

**Authors:** Bijaya Bahadur Thapa, Chen Huo, Rabin Budhathoki, Pratiksha Chaudhary, Soniya Joshi, Purna Bahadur Poudel, Rubin Thapa Magar, Niranjan Parajuli, Ki Hyun Kim, Jae Kyung Sohng

**Affiliations:** 1 Central Department of Chemistry, Tribhuvan University, Kirtipur 44618, Kathmandu, Nepal; bijaya.chem@gmail.com (B.B.T.); rabin.bc.992@gmail.com (R.B.); pratikshachaudhary274@gmail.com (P.C.); soniyajoshi157@gmail.com (S.J.); niranjan.parajuli@cdc.tu.edu.np (N.P.); 2 School of Pharmacy, Sungkyunkwan University, Suwon 16419, Republic of Korea; huochen_0213@163.com; 3 Institute of Biomolecule Reconstruction (iBR), Department of Life Science and Biochemical Engineering, Sun Moon University, Asan 31460, Republic of Korea; pbspoudel@gmail.com (P.B.P.); magarrubin@gmail.com (R.T.M.)

**Keywords:** antibiotics, microbial natural products, metabolomics, mass spectrometry

## Abstract

*Streptomyces* are well-known for producing bioactive secondary metabolites, with numerous antimicrobials essential to fight against infectious diseases. Globally, multidrug-resistant (MDR) microorganisms significantly challenge human and veterinary diseases. To tackle this issue, there is an urgent need for alternative antimicrobials. In the search for potent agents, we have isolated four *Streptomyces* species PC1, BT1, BT2, and BT3 from soils collected from various geographical regions of the Himalayan country Nepal, which were then identified based on morphology and 16S rRNA gene sequencing. The relationship of soil microbes with different *Streptomyces* species has been shown in phylogenetic trees. Antimicrobial potency of isolates was carried out against *Staphylococcus aureus* American Type Culture Collection (ATCC) 43300, *Shigella sonnei* ATCC 25931, *Salmonella typhi* ATCC 14028, *Klebsiella pneumoniae* ATCC 700603, and *Escherichia coli* ATCC 25922. Among them, *Streptomyces* species PC1 showed the highest zone of inhibition against tested pathogens. Furthermore, ethyl acetate extracts of shake flask fermentation of these *Streptomyces* strains were subjected to liquid chromatography-tandem mass spectrometric (LC-MS/MS) analysis for their metabolic comparison and Global Natural Products Social Molecular Networking (GNPS) web-based molecular networking. We found very similar metabolite composition in four strains, despite their geographical variation. In addition, we have identified thirty-seven metabolites using LC-MS/MS analysis, with the majority belonging to the diketopiperazine class. Among these, to the best of our knowledge, four metabolites, namely cyclo-(Ile-Ser), 2-n-hexyl-5-n-propylresorcinol, 3-[(6-methylpyrazin-2-yl) methyl]-1H-indole, and cyclo-(d-Leu-l-Trp), were detected for the first time in *Streptomyces* species. Besides these, other 23 metabolites including surfactin B, surfactin C, surfactin D, and valinomycin were identified with the help of GNPS-based molecular networking.

## 1. Introduction

Pathogens, which are causes of infectious diseases, acquire resistance to commonly used antibiotics due to excessive and improper application, leading to a growing necessity to combat the escalating issue of drug resistance. In 2015, the World Health Organization (WHO) officially recognized a worldwide health crisis, stating that 700,000 deaths occur annually due to infections caused by MDR. The WHO also projected a staggering 10 million deaths by the year 2050 if the issue is not effectively addressed [1].

For that reason, researchers around the world are currently focusing on the search for potent antimicrobials from natural sources with the help of different tools and techniques. Currently, metabolomics is emerging as a promising avenue for the analysis of the metabolites present in extracts under definite conditions and can facilitate and speed up the search for alternative bioactive molecules [2]. Metabolomics based on liquid chromatography-high resolution mass spectrometry (LC-HRMS) is an effective analytical approach for evaluating mixtures of metabolites in a biological system to aid in the dereplication and discovery of novel natural products [3]. In addition, a more recent approach in drug discovery programs is molecular networking, a tandem mass spectrometry (MS/MS) data organizational approach. It has been demonstrated that molecular networking can be used to analyze complex MS/MS data utilizing GNPS web-based platform, which can aid in the identification of various compounds because of the network formed due to similarity in collision-induced dissociation-tandem mass spectrometry (CID-MS/MS) fragmentation pattern [4]. This, in turn, contributes to the identification of precursor ions or molecules towards the discovery of lead compounds.

*Streptomyces*, the largest genus within the phylum *Actinomycetota*, is renowned for being a major source of a broad spectrum of bioactive secondary metabolites including antibiotics, anticancer and antiviral agents, and various enzymes [5]. These are usually isolated from the soil and marine environment using a variety of growth media, such as a series of International *Streptomyces* Project (ISP) media, casein starch agar (CSA) media, chitin agar, and starch nitrate agar. Earlier studies have reported that the genus *Streptomyces* has contributed to the production of more than 74% of the antibiotics currently available such as streptomycin, tetracycline, chloramphenicol, erythromycin, neomycin, nystatin, etc. [6,7].

The secondary metabolites produced from the *Streptomyces* species were mostly well known for their structural diversity and novelty. The search for novel lead compounds from conventional soil-isolated *Streptomyces* has experienced a downturn due to the rediscovery of the same or known compounds [8]. Despite this fact, some researchers have been reporting novel lead compounds from soil-derived *Streptomyces* species. Yang et al. isolated and identified eight new fasamycin-type antibiotics and streptovertimycins A–H from *Streptomyces morookaense* strain derived from soil [9]. Two new cyclic thiopeptides, geninthiocins E and F with antiviral activities were isolated from soil-derived *Streptomyces* sp. CPCC 200267 by Fang et al. [10]. Similarly, dipimprinine E and dipimprinine F two alkaloids showing significant anticancer activity were isolated from soil-derived *Streptomyces* sp. 44414B [11]. Picolinamycin, a novel antibiotic showing antibacterial activity against multi-drug resistant bacterial strains was isolated from soil-derived *Streptomyces* sp. SM01 [12]. Moreover, Hu et al. isolated and identified two new phenazines with antimicrobial activity; 6-hydroxyphenazine-1-carboxamide and methyl 6-carbamoylphenazine-1-carboxylate from soil-derived *Streptomyces* species [13]. In addition, a novel pranonaphthoquinone antibiotic, xiakemycin A, was isolated from *Streptomyces* sp. CC8-201 [14]. Thus, the above evidence indicates that there are still chances of finding new bioactive molecules from soil microbes.

Nepal’s varied ecological landscape spans a spectrum of climatic zones, stretching from tropical conditions in the lowlands to alpine environments in the lofty Himalayan peaks. This broad diversity of habitats provides a range of niches for microbial communities, including the genus *Streptomyces*. The capacity of *Streptomyces* species to generate a variety of physiologically active secondary metabolites, which are very powerful against microbial pathogens, makes them one of the most influential groups of bacteria [15]. In this study, we have collected four soil samples from different niches of Nepal with varying altitudes to isolate *Streptomyces* species. The primary goal of this study is to explore and analyze the potent antimicrobial metabolites present in *Streptomyces* isolated from soil by tandem mass spectrometry and GNPS-based molecular networking.

## 2. Results 

### 2.1. Isolation of Streptomyces Species 

Four soil samples PC1, BT1, BT2, and BT3 were collected from different ecosystems in Nepal, ranging from 1010 m to 2743 m above sea level (Table S1). As suggested by Bergey’s Manual of Systematic Bacteriology, bacterial strains PC1, BT1, BT2, and BT3 were initially identified based on biochemical assays, morphology, growth pattern, and Gram staining [16]. They were grown on ISP4 media, and their secondary metabolites harvesting was determined by the growth curve, which varied in individual bacterial strains. The color of aerial mycelia of the isolates BT1, BT2, and BT3 were greyish-white in appearance, while isolate PC1 was found to be whitish, which is presented in Figure S1. The Gram-staining revealed that the isolates were composed of hair-like mycelium and flagellated Gram-positive bacteria. We isolated more than twenty actinomycetes strains from each soil sample PC1, BT1, BT2, and BT3, respectively, but most of them were discarded based on their similar morphological characters, antimicrobial properties, and molecular sequencing. Finally, only one peculiar actinomycetes strain was selected from each soil sample. Further research on those soils may lead to the isolation of additional actinomycetes in the future.

### 2.2. PCR of 16S rRNA and Molecular Sequencing

About 1.5 kb 16S rRNA gene was amplified using universal oligonucleotides (27F and 1492R) from the genomic DNA of soil microbes (PC1, BT1, BT2, and BT3). Then, it was subjected to molecular sequencing as methods described earlier. The homology search using the BLAST tool in NCBI for the 16S rRNA gene sequences of these actinomycetes revealed that all their sequences exhibited high similarity (greater than 99.0%) with several sequences of *Streptomyces* species in the GenBank. Their 16S rRNA gene sequences were deposited in the GenBank (PC1, accession number OR577614; BT1, accession number OR578351; BT2, accession number PP106255; BT3, accession number OR905603). Multiple sequence alignment was performed for our sequences with twelve 16S rRNA genes showing the highest sequence similarity from NCBI, and a phylogenetic tree was generated using the MEGA software (version 11.0.13) (https://www.megasoftware.net/) [17]. All four were assigned as *Streptomyces* species considering their closeness to many sequences of different *Streptomyces.* Figure S3 displays the link between the isolates and the nearest phylogenetic neighbors as well as a comparison of the sequencing findings of *Streptomyces* isolates.

### 2.3. Antimicrobial Assays

The antimicrobial activity of *Streptomyces* species was tested against various Gram-positive and Gram-negative bacteria. The zone of inhibition of respective bacterial fermented extracts against tested pathogenic bacteria is listed below in Table S2. Among four isolates, *Streptomyces* sp. PC1 showed the highest zone of inhibition against all tested pathogenic bacteria. Thus, the MIC and MBC values (Table 1) of the extract of *Streptomyces* sp. PC1 was determined against *S. aureus* and *E. coli* along with *Streptomyces* species BT1, BT2, and BT3. The MICs of *Streptomyces* sp. PC1 against *S. aureus* and *E. coli* were 0.65 mg/mL and 1.5 mg/mL, respectively, while positive control neomycin exhibited MICs of 0.62 µg/mL and 0.78 µg/mL against *S. aureus* and *E. coli*, respectively. The MBCs of *Streptomyces* sp. PC1 against *S. aureus* and *E. coli* were 2.63 mg/mL and 3.0 mg/mL, respectively, compared to positive control (neomycin), which had MBCs of 1.25 µg/mL and 1.56 µg/mL against *S. aureus* and *E. coli*, respectively (Figure S2).

### 2.4. Metabolic Comparision in Streptomyces Species

Mass spectrometry generated raw data were analyzed through MestReNova software 12.0.0, Spain (https://mestrelab.com/, accessed on 10–30 December 2023). Each peak was analyzed, detected, aligned, and annotated. To visualize any difference in metabolites, the total ion chromatograms (TIC) of ethyl acetate (EA) extracts of *Streptomyces* species BT1, BT2, BT3, and PC1 were stacked together as shown in Figure 1. Finally, the results were compared with literature and database libraries. Thirty-seven metabolites were detected in all samples of classes such as diketopiperazine and alkyl resorcinol. The secondary metabolites annotated through LC-HRMS/MS analysis are shown in Table 2 and Figure S4. The base peak chromatograms (BPC) and MS profiles of identified metabolites are displayed in Supplementary Figures S5–S41.

A molecular ion at *m*/*z* 284.139 [M+H]^+^ detected at retention time 9.41 min was identified as brevianamide F, isolated previously from *Streptomyces* sp.TN262 [40]. Cyclo-(Phenylalanyl-Prolyl), previously isolated and identified in *Streptomyces* sp. [39], was also identified in this study at *m*/*z* 245.129 [M+H]^+^ as a protonated ion. Likewise, a molecular ion at *m*/*z* 261.123 [M+H]^+^ detected at retention time 6.59 min was annotated as maculosin, which was identified previously in *Streptomyces* sp. KTM18 [20]. We detected a molecular ion at *m*/*z* 171.113 [M+H]^+^ in retention time 6.46 min and identified it as cyclo-(Gly-Leu), already reported in *Streptomyces xanthophaeus* [49]. Cyclo-(d-Ala-l-Pro) was detected at a retention time of 2.94 min with *m*/*z* 169.097 [M+H]^+^, already identified in mangrove-derived *Streptomyces* sp. by Tan et al. [47]. Another cyclic dipeptide compound, cyclo-(Tyr-Val), was detected at *m*/*z* 263.141 [M+H]^+^ and reported previously in *Streptomyces* sp. [45]. Moreover, a precursor ion detected at *m*/*z* 295.147 [M+H]^+^ was identified as cyclo-(Phenylalanyl-Phenylalanyl), reported previously in *Streptomyces chrestomyceticus* [44]. Cyclo-(l-Leucyl-l-Leucyl), previously identified in *Streptomyces* sp. [42], was also identified in our study at *m*/*z* 227.176 [M+H]^+^ as a precursor ion. A molecular ion detected at *m*/*z* 219.114 [M+H]^+^ at retention time 7.97 min was identified as cyclo-(l-Phe-l-Ala), previously detected in *Streptomyces* sp. [38]. Moreover, a protonated ion detected at *m*/*z* 277.155 [M+H]^+^ was identified as cyclo(Tyr-Leu), previously identified from soil-derived *Streptomyces kunmingensis* [37]. In addition, *N*-phenethylacetamide was annotated for a molecular ion at *m*/*z* 164.107 [M+H]^+^. A precursor ion at *m*/*z* 425.233 [M+H]^+^ detected at a retention time of 14.02 min was identified as neomarinone [21]. Similarly, a molecular ion at *m*/*z* 245.129 [M+H]^+^ was identified as cyclo-(d-Pro-d-Phe), previously reported by Alshaibani et al. in *Streptomyces* sp. SUK 25 [54]. Based on the literature survey, cyclo-(l-Val-l-Leu) was identified at *m*/*z* 213.160 [M+H]^+^ as a protonated ion, already reported in *Streptomyces xiamenensis* MCC A01570 [19]. In addition, dibutyl phthalate was annotated for a molecular ion at *m*/*z* 279.160 [M+H]^+^. A precursor ion [M+H]^+^ at *m*/*z* 269.093 detected at retention time 14.38 min was putatively identified as 1-acetyl-3-methoxycarbonyl-β-carboline. This compound was previously isolated from the fungus *Ophiocordyceps sphecocephala* BCC 2661 [46].

A compound exhibiting a molecular ion peak at *m*/*z* 170.081 [M+H]^+^ was annotated as pyridoxine [23]. Furthermore, the precursor ion at *m*/*z* 245.10 [M+H]^+^ was annotated as cyclo-(l-Pro-l-OMet), detected at a retention time of 2.53 min, as per the analysis of spectral data of Yang et al. [24]. A molecular ion at *m*/*z* 1008.660 [M+H]^+^ detected at retention time 2.53 min was identified as surfactin C13 [25]. Likewise, a molecular ion at *m*/*z* 149.023 [M+H]^+^ detected at a retention time of 18.64 min was annotated as phthalic anhydride. Another molecular ion at *m*/*z* 556.531 [M+H]^+^ detected at a retention time of 20.68 min was identified as phytoceramide. Similarly, the [M+H]^+^ at *m*/*z* 155.081 [M+H]^+^ detected at retention time 2.20 min was identified as cyclo-(Pro-Gly), based on the existing literature [30]. A molecular ion at *m*/*z* 211.144 [M+H]^+^ detected at retention time 8.03 min was identified as cyclo-(l-Leu-l-Pro), previously reported by Zin et al. [31]. Furthermore, the molecular ion at *m*/*z* 261.119 [M+H]^+^ detected as a protonated ion at retention time 6.79 min was previously isolated from *Streptomyces asenjonii* by Abdelkader et al. and was annotated as cyclo-(l-Phenylalanyl-trans-4-hydroxy-l-Proline) [55]. As per the literature, the sodium adduct formed at *m*/*z* 344.183 [M+Na]^+^ detected at retention time 10.85 min was identified as coronafacoyl-l-isoleucine. This metabolite was previously isolated from *Streptomyces scabies* [56]. Another molecular ion peak detected at retention time 6.79 min having *m*/*z* 261.119 [M+H]^+^ was annotated as cyclo-(d-Pro-l-Tyr), previously detected at *Streptomyces* sp. strain 22-4 [32]. In addition, a metabolite was detected at *m*/*z* 197.12 [M+H]^+^ as a protonated ion in the retention time of 6.85 min, and identified as cyclo-(Pro-Val) as per the existing literature [34]. Similarly, the molecular ion detected at a retention time of 7.18 min with *m*/*z* 180.102 [M+H]^+^ was annotated as N-acetyl tyramine, from the spectral analysis carried out by Driche et al. [35]. The metabolite cyclo-(l-Ala-l-Leu), previously isolated from *Streptomyces* species from the soil environment, was detected at *m*/*z* 185.129 [M+H]^+^ as a protonated ion at the retention time of 7.57 min [57]. We identified another compound detected at *m*/*z* 274.275 [M+H]^+^ as *N*-lauryl diethanolamine. Likewise, a protonated ion detected at *m*/*z* 237.185 [M+H]^+^ at retention time 18.05 min was tentatively identified as 2-hexyl-5-methylresorcinol which was reported already in *S. clavuligerus* [53].

#### Identification of Metabolites New to *Streptomyces* Species

Among 37 metabolites, we identified four metabolites for the first time in *Streptomyces* species. A molecular ion [M+H]^+^ was detected at *m*/*z* 201.124 at a retention time of 6.20 min, with its MS^2^ peaks shown in Figure 2a.

The MS^2^ spectrum showed the fragment ions at *m*/*z* 173 [M+H-28]^+^ due to [C_8_H_17_N_2_O_2_]^+^ ion formed by loss of a CO molecule from precursor ion, *m*/*z* 114 [M+H-59-28]^+^ owing to [C_6_H_12_NO]^+^ ion formed by departure of a neutral C_2_H_5_NO unit and a CO molecule simultaneously, and [C_6_H_12_NO]^+^ ion further lose a CO molecule to give a distinct peak at *m*/*z* 86 attributed to C_5_H_12_N^+^ ion. Furthermore, an ion at *m*/*z* 60 [M+H-85-28-28]^+^ was observed due to the loss of a neutral C_5_H_11_N unit and two CO molecules as shown in Figure 3. Thus, we identified this compound as cyclo-(Ile-Ser), isolated previously from *Ophiocordyceps sobolifera* [48].

Another precursor ion was detected at a retention time of 12.09 min with *m*/*z* 224.118 [M+H]^+^. Its MS^2^ profile (Figure 2b) displayed a distinct peak at *m*/*z* 130.065 [M+H−94]^+^ due to the C_9_H_8_N^+^ ion attributed to the loss of a neutral C_5_H_6_N_2_ unit, as shown in Figure 4. Thus, this compound was putatively identified as 3-[(6-methylpyrazin-2-yl) methyl]-1H-indole, isolated and identified previously as a new alkaloid from marine *Serinicoccus profundi* sp. by Yang et al. [50]. To the best of our knowledge, this compound was observed for the first time in *Streptomyces* sp.

Furthermore, a molecular ion was observed at *m*/*z* 237.185 [M+H]^+^ in a retention time of 10.27 min, with its MS^2^ spectrum shown in Figure 5a.

The MS^2^ spectrum displayed a molecular ion peak [C_15_H_25_O_2_]^+^ as a base peak which may be due to incomplete fragmentation. Further, fragment ions were detected at *m*/*z* 124 [C_7_H_10_O_2_]^•+^ because of [M+H−C_5_H_11_−C_3_H_6_]^•+^ and *m*/*z* 137 owing to [M+H−C_5_H_11_−C_2_H_5_]^2•+^, as shown in Figure 6. Thus, this compound was tentatively identified as 2-n-hexyl-5-n-propylresorcinol. This alkyl resorcinol, previously reported in *Pseudomonas chlororaphis* PCL1606, has now been identified in *Streptomyces* sp. for the first time [52].

In addition, a protonated ion was detected at *m*/*z* 300.171 [M+H]^+^ in a retention time of 10.26 min. Its MS^2^ profile (Figure 5b) revealed a base peak with *m*/*z* 130 [M+H−170]^+^ due to the C_9_H_9_N^+^ ion formed by the loss of the C_8_H_14_N_2_O_2_ unit as shown in Figure 7. Thus, this compound was tentatively identified as cyclo-(d-Leu-l-Trp). Although this compound was previously reported in *Penicillium brevicompactum* [43], this is the first time it was observed in *Streptomyces* sp.

### 2.5. GNPS-Based Molecular Networking

To comprehensively investigate the detailed metabolite profile of four isolates, namely *Streptomyces* sp. BT1, BT2, BT3, and PC1, we conducted MS^2^ and GNPS metabolic profiling as illustrated in Figure 8. A total of 378 molecular ions were observed to have MS^2^ spectra of four samples represented by nodes that were connected by 788 edges in the molecular network. Out of these 378 ions, 62 ion pairs formed two-node clusters, while the remaining 335 molecular ions were self-looped. We successfully identified and dereplicated 25 known compounds through the GNPS library (Table 3), and you can find more detailed information on the GNPS website [58]. The GNPS approach revealed that the extracts from the four isolates consist of various compounds, including diketopiperazines, lipopeptides, dodecylamines, and tyramine alkaloids.

The extracts of the four samples (*Streptomyces* sp. BT1, BT2, BT3, and PC1) underwent metabolic profiling through MS^2^ data with positive ion mode and built a network using GNPS (Figure 9A). In Figure 9B, the ions with *m*/*z* 1022.680, 1036.690, and 1050.710 corresponded to surfactins B, C, and D, respectively. Intriguingly, the presence of an ion with *m*/*z* 1008.660, 16 Da lower than *m*/*z* 1022.680, suggests the removal of a methylene group from surfactin B, with a similar pattern observed for the ion at *m*/*z* 994.645 relative to *m*/*z* 1008.660, indicating structural similarities and related metabolic pathways. Notably, it was revealed that the BT1 isolate extract contained lipopeptides, as indicated by the base peak ion MS chromatograms. Additionally, Figure 9C revealed that the BT2 extract contained a significant amount of tyramine alkaloid, as suggested by the presence of annotated compounds isorugulosuvine and brevianamide F. Furthermore, the cluster associated with BT3 displayed an ammonium-adduct ion peak [M+NH_4_]^+^ at *m*/*z* 1128.670, corresponding to valinomycin (Figure 9D). In contrast to BT1, BT2, and BT3, PC1 was found to lack large molecules of lipopeptides. Instead, it contained smaller nitrogen-containing compounds, including pyridoxine, penciclovir, and β-indoleacetic acid (Figure 9E). These findings collectively highlight the diverse and distinct chemical profiles of the four isolates, shedding light on their unique metabolic pathways and chemical compositions.

## 3. Discussion

The improper usage and overprescription of pharmaceuticals have led to MDR pathogens, which have become a serious global issue [79]. Structural modification of a drug molecule is one adaptive strategy employed by bacteria to develop resistance to drugs. Thus, an alternative strategy to solve the problem of drug resistance could be the discovery of secondary metabolites that have the same therapeutic benefits and therefore can aid in the drug discovery program.

Soil ecosystem serves as key sources for *Streptomyces* isolation, exhibiting a broad range of metabolites production, with novel compounds emerging in response to varying nutritional or environmental factors [80]. Precise identification of bacterial isolates at the species level is essential, providing valuable insights into the microorganism, potential bioactive chemicals, and its distinctiveness [81]. Morphology also proves to be an essential factor in differentiating *Streptomyces* from other spore-forming actinomycetes, and in characterizing distinct species within the *Streptomyces* genus. Further, sequencing of 16S rRNA was performed for accurate identification of these strains suggesting that isolates are *Streptomyces* species including *Streptomyces* sp. BT1, *Streptomyces* sp. BT2, *Streptomyces* sp. BT3, and *Streptomyces* sp. PC1.

**Figure 1:**
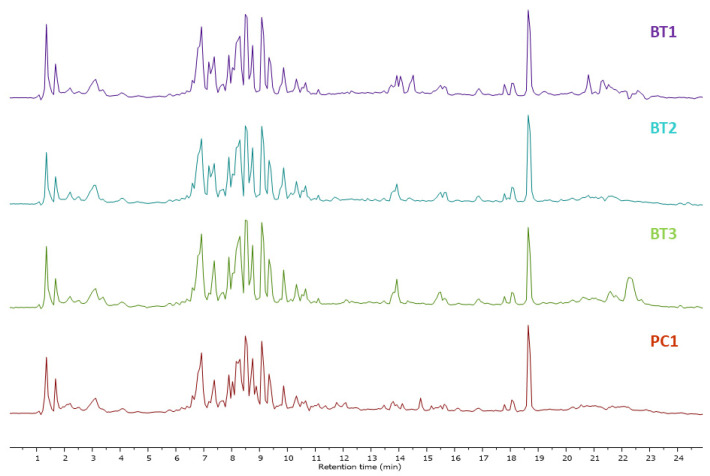
Total ion chromatograms obtained in EA extracts of *Streptomyces* species BT1, BT2, BT3, and PC1 in a stacked format.

**Figure 2:**
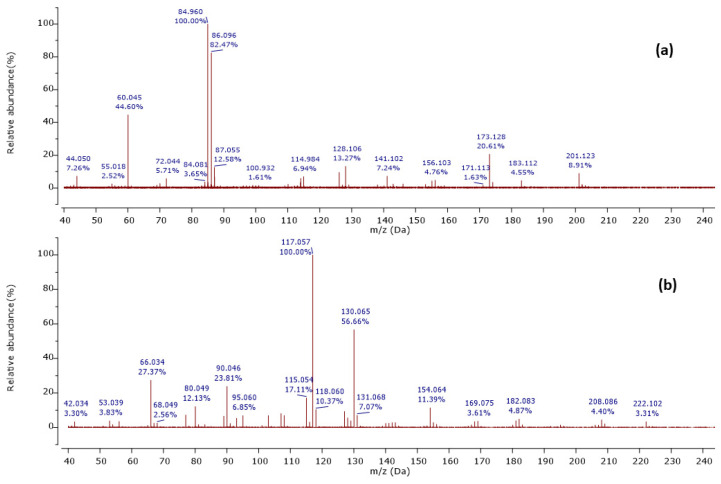
Observed MS/MS profiles of the precursor protonated molecules at *m*/*z* 201.124 (**a**) and *m*/*z* 224.118 (**b**).

**Figure 3:**
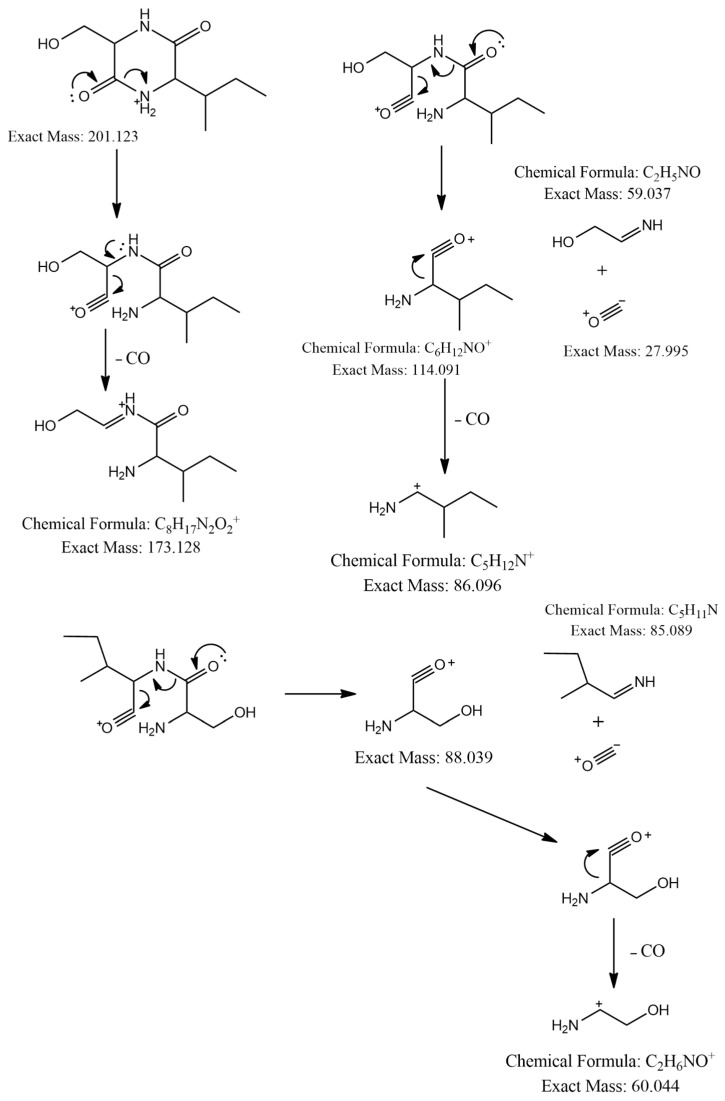
Observed fragmentation pattern of cyclo-(Ile-Ser) in (+)-ESI mode.

**Figure 4:**
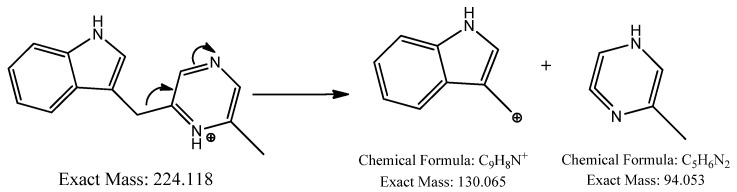
Observed fragmentation pattern of 3-[(6-methylpyrazin-2-yl) methyl]-1H-indole in (+)-ESI mode.

**Figure 5:**
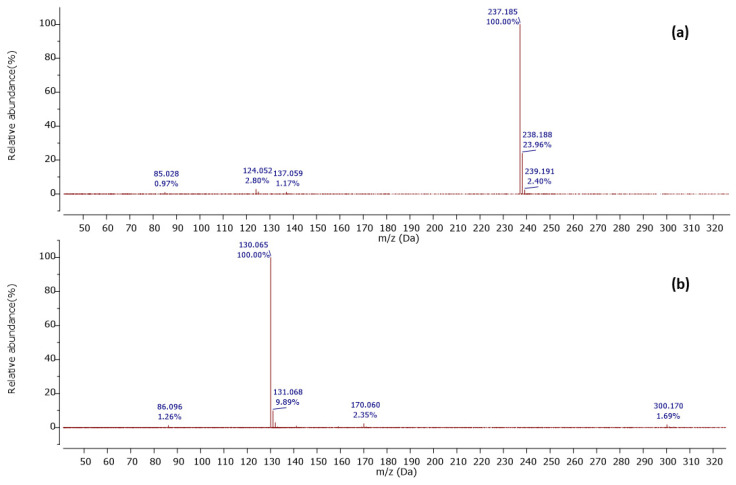
Observed MS/MS profiles of the precursor protonated molecules at *m*/*z* 237.185 (**a**) and *m*/*z* 300.170 (**b**).

**Figure 6:**
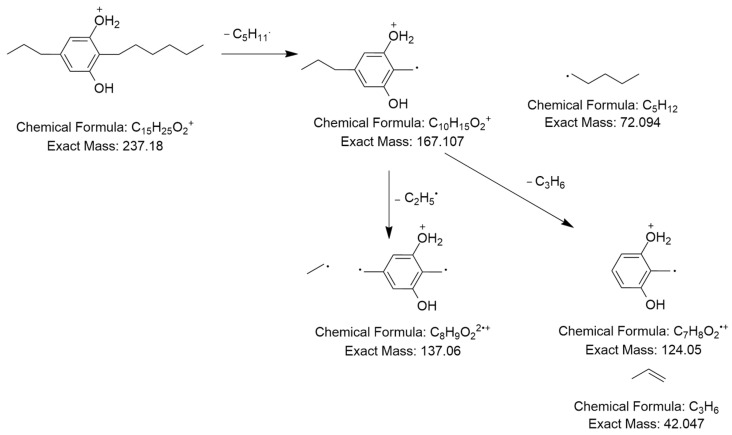
Observed fragmentation pattern of 2-n-hexyl-5-n-propylresorcinol in (+)-ESI mode.

**Figure 7:**
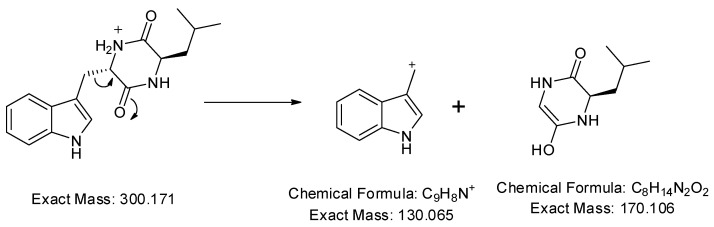
Observed fragmentation pattern of cyclo-(d-Leu-l-Trp) in (+)-ESI mode.

The total ion chromatogram of ethyl acetate extracts of *Streptomyces* sp. BT1, BT2, BT3, and PC1, shown in Figure 1 illustrated an identical chromatogram, signifying the elution of the same type of metabolites in the particular retention time. Despite originating from distinct ecological niches, we observed quite similar metabolic profiles across the species studied. This intriguing finding may be attributed to the uniformity in cultivation conditions experienced by all species. Specifically, each species was grown under an identical culture media, maintaining consistent temperature, and environmental conditions throughout the cultivation process. This standardized approach ensured that external factors did not introduce variability, allowing us to confidently attribute the observed similarities in metabolomic profiles to intrinsic biological factors shared among the studied species. Changes in the metabolites have been reported in *Streptomyces* species when altering the growth media. For instance, the use of four different media in the culture resulted in the generation of many bioactive compounds, specifically, three new macrolides were discovered when using a YMG agar medium, five additional polyketides from sterilized Waksman synthetic medium, and three newly discovered naphthomycins from oatmeal medium [82,83,84].

In our study, 37 metabolites were identified in *Streptomyces* sp. BT1, BT2, BT3, and PC1 using LC-HRMS/MS analysis in which many compounds belong to the diketopiperazine (DKP) class. DKP is a unique class of organic compounds resembling piperazine with two amide linkages, produced by microorganisms involved in fermentation processes, particularly crucial in the food and beverage industry. It has gained a massive interest nowadays due to its variety of biological activities. The majority of indole DKPs showed notable bioactivities, including cytotoxic, antibacterial, anti-inflammatory, and antiviral activities; as a result, these substances may serve as the basis for novel drug development [85]. Almost all the metabolites identified in this study demonstrate a potent antimicrobial effect as per the existing literature, leading the bacterial strain towards a good antimicrobial activity. Cyclo-(d-Pro-d-Phe) showed antimicrobial activity against phytopathogenic *Rhodococcus fascians* [86]. Similarly, cyclo-(l-Pro-l-OMet) demonstrated antimicrobial activity against *Escherichia coli*, *Pseudomonas aeruginosa*, and *Staphylococcus aureus* showing a 100 µg/mL MIC value along with antifungal activity with a 50 µg/mL MIC value [87]. Due to the potent antimicrobial activity and synergistic impact in the development of *enterococci*, which are resistant to vancomycin, with MIC between 0.25 and 0.5 µg/mL, cyclo-(l-Leu-l-Pro) has a wide therapeutic application [88]. The ability to demonstrate antimicrobial activity against *Vibrio anguillarum* with a MIC value of 2.68 × 10^−7^ µg/mL makes cyclo-(l-Phenylalanyl-trans-4-hydroxy-l-Proline) a successful candidate that might show therapeutic applications [89]. Proline-rich antimicrobial peptides, or PrAMPs, have a significant amount of antibacterial activity and minimal cytotoxicity, making them prospective agents against infections that are resistant to several drugs [90]. Likewise, cyclo-(d-Pro-l-Tyr) is reported to demonstrate a potential antimicrobial effect against various plant pathogenic bacteria [32]. Cyclo-(Pro-Val) exhibited an antimicrobial effect against *Vibrio anguillarum* with a MIC value of 7.14 × 10^−7^ µg/mL [89], but this metabolite did not stop the proliferation of cancer cells. Another metabolite *N*-acetyltyramine detected in this work highlights a great potential to be used as a successful candidate for further study against drug-resistant bacteria as it shows a MIC value of 30 mg/mL [35]. A cyclic dipeptide called maculosin, which was isolated from *Pseudomonas rhizosphaerae*, has been shown to have antibacterial properties against a range of marine bacteria, including *Bacillus cereus*, *Ruegeria* sp., and *Pseudoalteromonas piscida* [91]. Strong antibacterial action (26–37 µg/mL) was demonstrated by a glycoside of maculosin that was isolated from marine *Streptomyces* sp. ZZ446, against methicillin-resistant *Staphylococcus aureus*, *Escherichia coli*, and *Candida albicans* [20]. Hence, most of the diketopiperazines identified in our study might be responsible for the observed antimicrobial potential.

Earlier research indicates that brevianamide F could potentially be employed in the treatment of cardiovascular dysfunction, and bacterial infection [92,93]. Apart from functioning as a glycopeptide-like antibiotic, cyclo-(Phenylalanyl-Prolyl) has demonstrated various significant roles, including inhibiting membrane permeability and decelerating DNA synthesis [94]. Due to its potent antioxidant capabilities and non-toxic nature, maculosin could be a promising candidate for diverse applications in cosmetics and therapeutics [20]. Prior research revealed that cyclo-(Gly-Leu) interacts with dopamine receptors, suggesting a potential involvement of central dopamine receptors in the pathophysiology of hypertension [95]. However, cyclo-(d-Ala-l-Pro) isolated from the fungus *Colpoma* sp. was reported to show poor antimicrobial activity [96]. It was reported that at an optimal concentration of 10 μg/mL, cyclo-(Tyr-Leu) was able to enhance the mycelial growth of *H. marmoreus* [97]. It was reported that *N*-phenethylacetamide hindered the TGF-β/Smad pathway, restraining the metastasis of A549 cells by impacting TGF-β-induced epithelial-mesenchymal transition (EMT) [98]. Further, cyclo-(l-Ala-l-Leu) develops disease resistance contrary to *Pseudomonas syringae* attack, but it does not directly stop the growth of fungi [99].

As per the existing literature, surfactin C13 demonstrated cytotoxic potential against various cancer cell lines [100]. One of the most common uses of phthalic anhydride is the production of phthalate esters, which can be used as plasticizers [101]. Phytoceramide is reported to promote hydration and enhance the healing process of damaged human stratum corneum in human skin and can be used in the cosmetic industry for creating skin barrier moisturizers [102]. Moreover, phytoceramides were found to be cytotoxic to the MES-SA, MCF-7, and HK-2 cell lines in the previous study of *Monanchora clathrata*, and have further proven to be used in the prevention of neurodegeneration in both in vivo and in vitro [103]. The ability of cyclo-(Pro-Gly) to decrease motor neuronal death demonstrates the neuroprotective function after brain injury and this compound also shows anxiolytic activity [104,105]. Similarly, coronafacoyl-l-isoleucine is a biosynthetic intermediate of coronatine, which seems to amplify the degree of illness symptoms brought on by pathogenic microorganisms during host infection [106,107]. *N*-lauryl diethanolamine is a plastic antistatic agent with high proton affinity, thus detected in positive ionization mode and reported as an interference substance that leaches from plastic microtubes during sample pretreatment [51]. Neomarinone was reported to show in vitro cytotoxicity, with an IC_50_ value of 8 µg/mL against HCT-116 colon cancer cells [21]. Cyclo-(d-Pro-d-Phe) has been reported to show antifungal, quorum sensing, and antimicrobial activities, and it does not show any toxicity effect up to 200 µg/mL to human cell lines [108]. Cyclo-(l-Val-l-Leu) metabolite shows a 50% inhibition rate against PANC-1 and Hela S3 cancer cells [109]. Dibutyl phthalate demonstrated a toxic effect in humans causing headaches and vertigo and could affect the throat and nose severely. Furthermore, this compound has long-term negative effects on the developing fetus and testicles [110,111]. However, despite all the negative effects, it does show antimicrobial and antifungal activities [112]. Dibutyl phthalate isolated from *Streptomyces albidoflavus* exhibited potent antimicrobial activity against both Gram-positive and Gram-negative bacteria [22]. The ability of pyridoxine to maintain the proper ratio of potassium and sodium in the body followed by enhancement in the production of red blood cells aids in the prevention of homocysteine synthesis and develops immunity against cancer [113]. 

In general, compounds of class β-carboline are known to have biological activities including antiviral, antibiotic, anticancer, and antimalarial properties [114,115]. Similarly, alkyl resorcinol, an important structural group of amphiphilic phenolic lipids, is reported to show a variety of biological activities, such as cytotoxic, genotoxic, antioxidant, and signaling capabilities [116]. 2-n-hexyl-5-n-propylresorcinol (HPR) identified in our study is reported to demonstrate antimicrobial properties against both fungi and bacteria [117]. HPR is a tiny chemical produced by several bacteria from the cell that exhibits some antibacterial action in the surroundings [52]. In our research, we identified cyclo-(d-Leu-l-Trp) in *Streptomyces* sp. for the first time, and researchers have reported that it could enhance the root growth of seedlings [43]. However, 3-((6-methylpyrazin-2-yl)methyl)-1H-indole was reported to show a low cytotoxicity effect against human liver cell lines and demonstrated poor antibacterial activity [50]. Hence, previously reported activities of compounds have shown the factor responsible for those antimicrobial activities, and further studies on these annotated compounds can lead to the discovery of novel antimicrobials.

**Figure 8:**
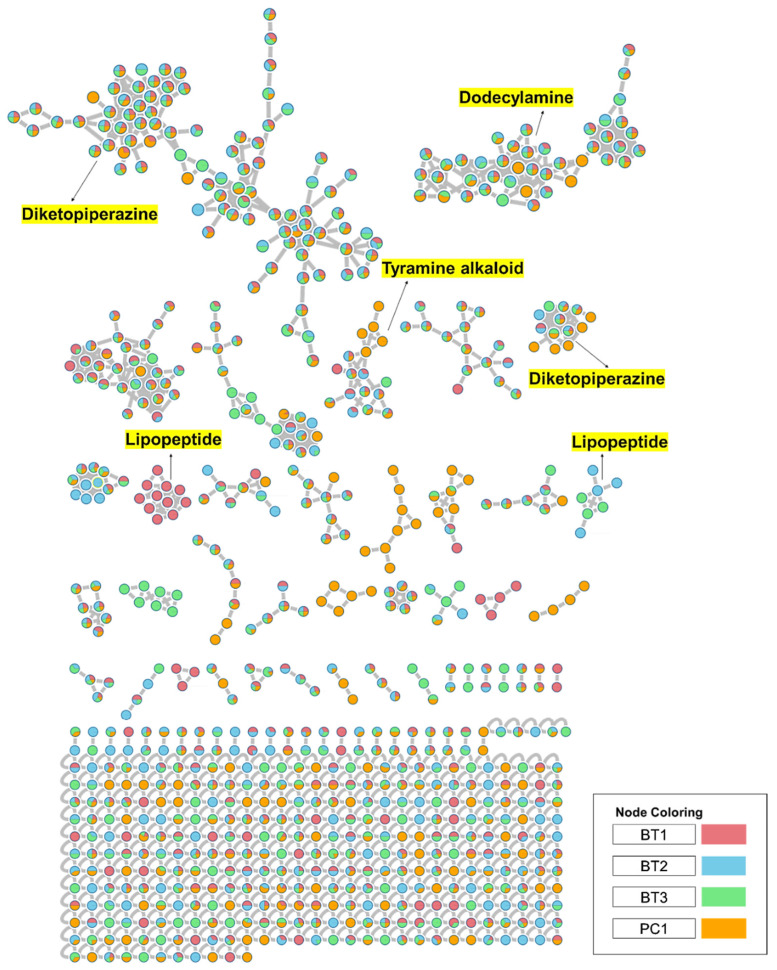
Full molecular networking was created using MS/MS data in positive mode from extracts of *Streptomyces* species BT1, BT2, BT3, and PC1. Nodes are labeled with parent mass. The networking is displayed as a pie chart with pink, blue, green, and orange indicating the chemical composition distribution of *Streptomyces* species BT1, BT2, BT3, and *Streptomyces* sp.PC1 extracts, respectively.

**Figure 9:**
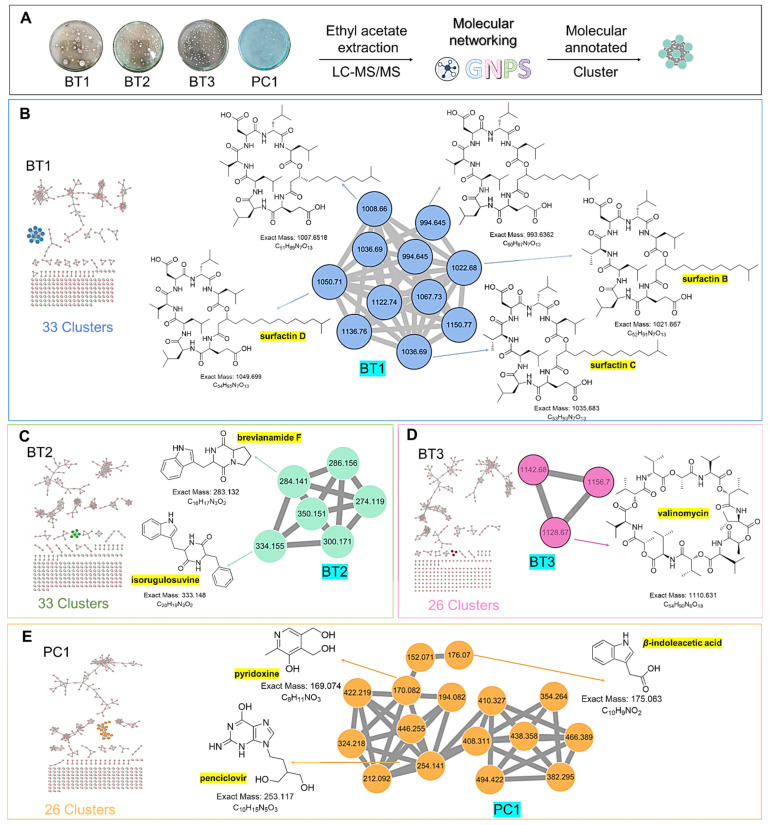
(**A**) Molecular networking analysis and identification of compounds in four samples. (**B**) Zoomed-in molecular networking of annotated compounds from BT1. (**C**) Zoomed-in molecular networking of annotated compounds from BT2. (**D**) Zoomed-in molecular networking of annotated compounds from BT3. (**E**) Zoomed-in molecular networking of annotated compounds from PC1.

Furthermore, GNPS-based molecular networking was employed for the molecular annotation of metabolites in addition to manual interpretation of LC-MS/MS data. GNPS is a web-based mass spectrometry platform accessible to the public and offers several tools for analyzing MS/MS data. This platform enables the annotation of compounds either by using spectral library search or molecular networking-based grouping of compounds into families or clusters of molecules [92]. Different compounds were annotated from GNPS including different classes’ majority of diketopiperazines, lipopeptides, dodecyl amine, and tyramine alkaloids (Figure 8). In the manual interpretation of MS/MS data, we used several natural product-based databases. Whereas, GNPS uses its library and some other reference libraries for spectral hitting, and approximately 1.8% to 2% of metabolites are annotated in untargeted metabolomics experiments due to a lack of sufficient chemical space coverage [118,119]. Moreover, ions with very low intensity lying near noise level are generally neglected in manual interpretation but GNPS workflow utilizes all ions including ions with low intensity for spectral hitting. Thus, these factors may be responsible for the variation in the number and metabolites detected in manual interpretation and GNPS spectral library hitting. 3-epi-xestoaminol C, a stereoisomer of xestoaminol C, was identified via molecular networking. This compound was reported to demonstrate IC_50_ values of 19.4 μM against *M. tuberculosis* H37Ra, 8.8 μM against HL-60 cells, and 18.0 μM against HEK cells, as reported in the literature [59]. These results emphasize the varied and distinct metabolite profiles of four isolates, providing insights into their metabolic pathways, chemical compositions, and bioactivities.

## 4. Materials and Methods

### 4.1. Isolation and Characterization of Streptomyces Species

Soil samples were collected from various ecosystems ranging up to 2743 m, altitudes in Nepal. The soils were taken from a depth of 10–15 cm beneath the earth’s surface representing diverse sampling habitats including agriculture fields, forests, and hilly regions (Table S1). The collected soil samples were kept in a sterilized zip bag and stored at 4 °C in the laboratory.

Isolation of *Streptomyces* species was carried out using the spread plate technique developed by William and Davies, amended with antibiotics supplements [120]. One gram of each soil sample was dissolved in 9 mL of distilled and autoclaved water and thoroughly mixed by using a vertex shaker. A three-fold serial dilution was carried out to lower the bacterial population. Then, 100 µL of each serially diluted soil suspension was spread over the ISP4 medium with the help of a sterile glass spreader. 20 mg/L nalidixic acid and 50 mg/L cycloheximide were also added to the ISP4 medium to inhibit Gram-negative bacteria and fungus species, respectively. Finally, the plates were incubated at 28 °C for 7 days [121]. 

### 4.2. Genomic DNA Extraction and 16S rRNA Gene Sequencing

The genomic DNA of *Streptomyces* species was isolated using the phenol-chloroform method as described in the standard protocol of molecular biology [122]. For taxonomy identification, 16S rRNA gene was amplified using oligonucleotides 27F: 5′-AGAGTTTGATCCTGGCTCAG-3′, and 1492R: 5′-GGTTACCTTGTTACGACTT-3′. The Polymerase Chain Reaction (PCR) was carried out in a 50 μL reaction mixture using 5x*Taq*-PCR Premix (GenoTech Corporation, Daejeon, Republic of Korea) containing *Taq*-polymerase within 30 cycles. A cycle was programmed with denaturation at 98 °C (10 s), an annealing at 54 °C (10 s), and an extension at 72 °C (2 min). The PCR products were isolated from low-melting agarose gel and were purified by using the QIAquick Gel Extraction Kit (Qiagen, Germantown, MD, USA). The purified PCR products were sequenced using the same primers (27F and 1492F) through the Sangar dideoxy method by GenoTech Co., Daejeon, Republic of Korea. Then, the BLAST tool of the National Center for Biotechnology Information (NCBI) was used to compare the 16S rRNA gene sequences of our isolates with those in the GenBank database [123]. Then, 12 sequences that were highly similar to our amplified 16S rRNA genes were subjected to multiple sequence alignment with our sequences, followed by the generation of a phylogenetic tree using the neighbor-joining method with the MEGA Software (version 11.0.13) (https://www.megasoftware.net/) [17].

### 4.3. Fermentation and Extraction of Metabolites

The seed culture of *Streptomyces* species was carried out in Tryptic Soy Broth (TSB) medium (Tryptone 17.0 g, Soytone 3.0 g, Glucose 2.5 g, Sodium Chloride 5.0 g, Dipotassium Phosphate 2.5 g, pH 7.3 ± 0.2 at 28 °C; volume 1 L water). After sufficient growth of *Streptomyces* species, 1 mL (1%) of bacterial suspension was transferred into 100 mL of freshly prepared TSB medium for fermentation (for production of secondary metabolites). The incubation was conducted at 28 °C for 5–7 days at 180 rpm in a shaking incubator until bacterial growth reached the stationary phase. Secondary metabolites were harvested by mixing an equal volume of ethyl acetate with culture broth. The clear supernatant was transferred into a clean and dry beaker and evaporated in a water bath at 37 °C for 2–3 days to obtain the crude bacterial extracts. Then, the dried extract was placed at 4 °C until use [124].

### 4.4. Antimicrobial Assays

The primary screening of the isolates was carried out in Mueller Hinton Agar (MHA) medium by a perpendicular streaking method. The agar-well diffusion method was used for the secondary screening of crude extracts [125]. In this method, the standard culture of test organisms was swabbed over the MHA medium with the help of sterile cotton buds. For this, Gram-positive pathogen *Staphylococcus aureus* ATCC 43300, and various Gram-negative pathogens, *Shigella sonnei* ATCC 25931, *Salmonella typhi* ATCC 14028, *Klebsiella pneumoniae* ATCC 700603, and *Escherichia coli* ATCC 25922 were tested. These tested pathogens were incubated in Mueller Hinton Broth (MHB) medium at 37 °C for 24 h. Then, their turbidity was adjusted to that of standard 0.5 McFarland (1.5 × 10^8^ CFU/mL) for further use. Then, the wells were made with the help of sterile cork borers of 6 mm in diameter. Finally, the wells were filled with the positive control (1 mg/mL neomycin), negative control (50% DMSO), and a working solution of extract dissolved in 50% DMSO. At last, the plates were incubated at 37 °C for 24 h and observed for the clear zone of inhibition.

The MIC and MBC of the crude extract were determined by the broth microdilution method according to the Clinical Laboratory Standard Institute (CLSI) [126]. A series of two-fold dilutions of extract were prepared directly in sterile 96-well microdilution plates containing MHB to obtain a range of concentrations. The bacterial inoculum was added at a final concentration of 1.5 × 10^8^ CFU/mL by diluting 1:100 after matching the turbidity of the 0.5 McFarland turbidity culture in MHB. Finally, 30 μL of bacteria were added to each well except for the negative control. The MBC was determined by streaking the good contents onto nutrient agar plates, followed by incubation for over 18 h at 37 °C [127]. 

### 4.5. Metabolic Comparison

Ethyl acetate (EA) extracts of *Streptomyces* species were then subjected to liquid chromatography-high resolution tandem mass spectrometric analysis (LC–HRMS/MS) by employing an Agilent G6545B quadrupole time-of-flight (Q-TOF) mass spectrometer (Agilent Technologies, Santa Clara, CA, USA) equipped with a heated electrospray ion source at Sungkyunkwan University, Suwon, Republic of Korea. For MS/MS analysis, four samples (BT1, BT2, BT3, and PC1) were prepared by dissolving EA extracts in HPLC-grade solvent (methanol) at a concentration of 1 mg/mL. A volume of 150 μL from each sample was transferred to HPLC autosampler vials. Chromatographic separation was achieved using an Acquity^®^ (Indio, CA, USA) UPLC BEH reverse-phase C18 column (150 mm × 2.1 mm, 1.7 μM). The mobile phases, acidified with 0.1% formic acid, consisted of H_2_O (A) and acetonitrile (B). The composition of the organic solvent was used as follows: 5% from 0.00 to 2.00 min, 20% at 5.00 min, 100% at 20.00 min, and then returning to 5% from 23.00 to 25.00 min. The injection volume for each sample was maintained at 3 μL, and a constant flow rate of 0.5 mL/min was maintained. The MS/MS data acquisition was performed using the electrospray ionization (ESI) technique in positive ion mode with a *m*/*z* range of 50–1200 Da, collision energies set at 15 V and 40 V, and a full width at half maximum (FWHM) of 3000.

The raw data (.d format) were converted into .mzXML format [128] and further annotated using CSI: FingerID, which is a graphical interface incorporated in SIRIUS software (version 5.8.0) [129]. The calculated mass, absolute error, RDBE, and molecular formulae were generated by MestReNova software (version 12.0.0) (accessed on 10–30 December 2023) and were compared with the formula generated by SIRIUS. Furthermore, the annotated compounds were validated via the literature survey using SciFinder (https://scifinder-n.cas.org/, accessed on 12–14 January 2024), and natural products-based databases such as PubChem (https://pubchem.ncbi.nlm.nih.gov/, accessed on 25–30 December 2023), ChemSpider (https://www.chemspider.com/, accessed on 25–30 December 2023), Natural Products Atlas (https://www.npatlas.org/, accessed on 25–30 December 2023), LOTUS (https://lotus.naturalproducts.net/, accessed on 25–30 December 2023), and libraries search using SIRIUS software (version 5.8.0). SIRIUS score, generated by software, serves as a parameter for gauging the confidence of molecular annotation, with a higher score indicating greater confidence in the annotation.

### 4.6. GNPS-Based Molecular Networking Analysis

EA extracts of four *Streptomyces* species were prepared for MS/MS analysis by dissolving them in HPLC-grade MeOH (1 mg/mL), with 150 μL of each sample transferred to an HPLC autosampler vial. Metabolomic profiling was conducted using an Agilent G6545B quadrupole time-of-flight (Q-TOF) mass spectrometer (Agilent Technologies, Santa Clara, CA, USA) equipped with a heated electrospray ion source (HESI). Chromatographic separation was achieved using an Acquity^®^ UPLC BEH reverse-phase column C18 (150 mm × 2.1 mm, 1.7 μM). The mobile phase consisted of 0.1% formic acid in H_2_O (A), and acetonitrile (B) in varying proportions: 5% (B) from 0 to 2 min, 5–20% (B) from 2 to 5 min, 20–100% (B) from 5 to 20 min, 100% (B) from 20 to 23 min, and 100–5% (B) from 23 to 25 min. Each sample was injected at a volume of 3 μL, with a flow rate of 0.3 mL/min maintained. MS/MS analysis was conducted using electrospray ionization (ESI) in positive ion mode. Spectral hits were performed using a modified version with an *m*/*z* range of 50–1700, with collision energies set at 15 V and 40 V, capillary voltage (2.5 kV), and a full width at half maximum (FWHM) of 3000 [129]. The raw data ‘.d format’ files were first converted to ‘.mzXML’ format using open-source MSConvert software (https://proteowizard.sourceforge.io/download.html) (Version: 3.0). To upload the files, the recommended FTP client WinSCP was utilized, and they were transferred to the GNPS platform. Visualizing the MS/MS data followed established GNPS-based procedures (accessed on 17 January 2023). Molecular networks generated in GNPS were further exported to Cytoscape (version 3.9.1.) in ‘.graphml’ format to enable customized visualization and additional analysis.

## 5. Conclusions

*Streptomyces* genus can produce a wide range of bioactive secondary metabolites that have good efficacy against several MDR pathogens. In this study, *Streptomyces* species were isolated from the soils collected from various habitats in Nepal and characterized by using 16S rRNA gene sequencing. Further, the ethyl acetate extracts of *Streptomyces* sp. were subjected to LC-HRMS/MS analysis and GNPS-based molecular networking. We found a similar metabolite profile in the mentioned species. Thirty-seven different secondary metabolites encompassing a range of compounds, including polypeptides, bacterial alkaloids, amino compounds, and diketopiperazines were annotated in our study. These metabolites, specifically diketopiperazines are reported to demonstrate an effective antimicrobial activity based on existing literature. In addition, to the best of our knowledge, four metabolites, namely cyclo-(Ile-Ser), 2-n-hexyl-5-n-propylresorcinol, 3-(6-methylpyrazin-2-yl) methyl)-1H-indole and cyclo-(d-Leu-l-Trp) were reported for the first time in *Streptomyces* species. In addition, these findings contribute new additional value by revealing new sources for the isolation of these metabolites, which can aid in the drug discovery process. Furthermore, despite being isolated from different ecological niches, we observed similar metabolome profiles in the studied species. These results could be supported by the fact that all the species were cultivated under identical culture media, temperature, and conditions. Therefore, further study can be conducted on the *Streptomyces* species by varying fermentation media and culture conditions to unveil new metabolites. Moreover, the newly annotated metabolites in this study can further be validated through NMR, followed by their biological evaluation, which could facilitate the drug discovery process.

## Figures and Tables

**Table 1:** MIC and MBC of various extracts against *Staphylococcus aureus* and *Escherichia coli*.

*Streptomyces* Species Extracts	BT1 (mg/mL)	BT2 (mg/mL)	BT3 (mg/mL)	PC1 (mg/mL)	Neomycin (µg/mL)
MIC (*Staphylococcus aureus*)	2.295	1.960	1.875	0.658	0.625
MIC (*Escherichia coli*)	1.090	1.738	0.937	1.500	0.781
MBC (*Staphylococcus aureus*)	4.590	3.920	3.750	2.635	1.250
MBC (*Escherichia coli*)	2.188	3.477	1.875	3.000	1.562

**Table 2:** The list of annotated compounds in ethyl acetate fermentation extracts of *Streptomyces* species BT1, BT2, BT3, and PC1.

C.N	Annotated Compound	Exact Mass *m*/*z*	Observed Mass *m*/*z*	Detected Ion	Molecular Formula	RDBE	Absolute Error (ppm)	RetentIon Time (min)	Bacterial Source	CSI:FingerID Score (%)	References
1	Cyclo-(d-Pro-d-Phe)	244.120	245.129	[M+H]^+^	C_14_H_16_N_2_O_2_	8.0	3.20	8.76	BT1	99.24	[18]
2	Cyclo-(l-Val-l-Leu)	212.159	213.160	[M+H]^+^	C_11_H_20_N_2_O_2_	3.0	1.84	9.34 (BT1)	BT1, BT2	92.71	[19]
3	Maculosin	260.115	261.123	[M+H]^+^	C_14_H_16_N_2_O_3_	8.0	0.57	6.59 (BT1)	PC1, BT1, BT3	98.00	[20]
4	Neomarinone	424.225	425.233	[M+H]^+^	C_26_H_32_O_5_	11.0	4.70	14.02	BT1	48.47	[21]
5	Dibutyl phthalate	278.152	279.160	[M+H]^+^	C_16_H_22_O_4_	6.0	2.18	18.64 (BT1)	BT1, BT2	95.91	[22]
6	Pyridoxine	169.073	170.081	[M+H]^+^	C_8_H_11_NO_3_	4.0	1.34	1.35 (BT1)	BT1, BT2	99.45	[23]
7	Cyclo-(l-Pro-l-OMet)	244.087	245.10	[M+H]^+^	C_10_H_16_N_2_O_3_S	4.0	0.70	2.53	BT2	76.71	[24]
8	Surfactin C13	1007.651	1008.660	[M+H]^+^	C_51_H_89_N_7_O_13_	11.0	3.394	21.29	BT1	99.72	[25]
9	Cyclo-(l-Valyl-l-Phenyl alanyl)	246.136	247.144	[M+H]^+^	C_14_H_18_N_2_O_2_	7.0	0.74	9.87	BT2	93.04	[26]
10	Di-*n*-butyl terephthalate	278.151	279.159	[M+H]^+^	C_16_H_22_O_4_	6.0	1.76	18.58	BT3	54.11	[27]
11	Phthalic anhydride	148.015	149.023	[M+H]^+^	C_8_H_4_O_3_	7.0	0.63	18.64	BT3	99.24	[28]
12	Phytoceramide	555.522	556.531	[M+H]^+^	C_34_H_69_NO_4_	11.0	1.22	20.68	BT3	71.70	[29]
13	Cyclo-(Pro-Gly)	154.073	155.081	[M+H]^+^	C_7_H_10_N_2_O_2_	4.0	3.27	2.20 (BT3)	PC1, BT1, BT2, BT3	97.50	[30]
14	Cyclo-(l-Leu-l-Pro) or Gancidin W	210.136	211.144	[M+H]^+^	C_11_H_18_N_2_O_2_	4.0	1.41	8.03 (BT2)	PC1, BT1, BT2, BT3	98.69	[31]
15	Cyclo-(l-Phenylalanyl-*tran*s-4-hydroxy-l-Proline)	260.112	261.119	[M+H]^+^	C_14_H_16_N_2_O_3_	8.0	1.32	6.72 (PC1)	PC1, BT1, BT2, BT3	62.83	[32]
16	Coronafacoyl-l-isoleucine	321.194	344.183	[M+Na]^+^	C_18_H_27_NO_4_	6.0	0.02	10.85 (PC1)	PC1, BT3	36.16	[33]
17	Cyclo-(d-Pro-l-Tyr)	260.112	261.119	[M+H]^+^	C_14_H_16_N_2_O_3_	4.0	1.85	6.79 (BT2)	PC1, BT1, BT2, BT3	87.08	[32]
18	Cyclo-(Pro-Val)	196.120	197.129	[M+H]^+^	C_10_H_16_N_2_O_2_	4.0	2.10	6.59 (BT2)	PC1, BT1, BT2, BT3	95.58	[34]
19	*N*-Acetyltyramine	179.094	180.102	[M+H]^+^	C_10_H_13_NO_2_	5.0	2.48	7.18 (BT2)	PC1, BT1, BT2, BT3	73.66	[35]
20	Cyclo-(l-Ala-l-Leu)	184.120	185.129	[M+H]^+^	C_9_H_16_N_2_O_2_	3.0	1.25	7.57 (BT2)	BT1, BT2, BT3	97.87	[36]
21	Cyclo-(Tyr-Leu)	276.147	277.155	[M+H]^+^	C_15_H_20_N_2_O_3_	7.0	1.07	7.84 (BT2)	PC1, BT1, BT2, BT3	94.12	[37]
22	Cyclo-(l-Phe-l-Ala)	218.104	219.114	[M+H]^+^	C_12_H_14_N_2_O_2_	7.0	4.91	7.97 (BT2)	PC1, BT1, BT2, BT3	95.63	[38]
23	Cyclo-(Phenylalanyl-Prolyl)	244.121	245.129	[M+H]^+^	C_14_H_16_N_2_O_2_	8.0	1.00	8.69 (BT2)	PC1, BT1, BT2, BT3	99.62	[39]
24	Brevianamide F or Cyclo-l-Trp-l-Pro	283.131	284.139	[M+H]^+^	C_16_H_17_N_3_O_2_	10.0	0.47	9.41 (BT2)	PC1, BT1, BT2, BT3	99.40	[40]
25	*N*-Phenethylacetamide	163.099	164.107	[M+H]^+^	C_10_H_13_NO	5.0	1.63	9.74 (BT2)	PC1, BT1, BT2, BT3	99.24	[41]
26	Cyclo-(l-Leucyl-l-Leucyl)	226.168	227.176	[M+H]^+^	C_12_H_22_N_2_O_2_	3.0	0.83	10.32 (BT2)	PC1, BT1, BT2, BT3	94.97	[42]
27	Cyclo-(d-Leu-l-Trp)	299.163	300.171	[M+H]^+^	C_17_H_21_N_3_O_2_	9.0	1.61	10.26 (BT2)	PC1, BT1, BT2, BT3	85.35	[43]
28	Cyclo-(Phenylalanyl-Phenylalanyl)	294.139	295.147	[M+H]^+^	C_18_H_18_N_2_O_2_	11.0	1.55	11.11 (BT2)	PC1, BT1, BT2, BT3	69.32	[44]
29	Cyclo-(Tyr-Val)	262.133	263.141	[M+H]^+^	C_14_H_18_N_2_O_3_	7.0	0.27	7.71 (BT2)	BT1, BT2, BT3	76.92	[45]
30	1-Acetyl-3-methoxycarbonyl-β-carboline	268.086	269.093	[M+H]^+^	C_15_H_12_N_2_O_3_	11.0	1.95	14.38 (BT2)	BT1, BT2	81.21	[46]
31	Cyclo-(d-Ala-l-Pro)	168.089	169.097	[M+H]^+^	C_8_H_12_N_2_O_2_	4.0	0.56	2.99 (BT2)	PC1, BT1, BT2, BT3	98.56	[47]
32	Cyclo-(Ile-Ser)	200.125	201.124	[M+H]^+^	C_9_H_16_N_2_O_3_	3.0	1.02	6.20 (PC1)	PC1, BT1, BT2, BT3	83.49	[48]
33	(*S*)-3-Isobutylpiperazine-2,5-dione or Cyclo(Gly-Leu)	170.105	171.113	[M+H]^+^	C_8_H_14_N_2_O_2_	3.0	1.11	6.46 (BT2)	PC1, BT1, BT2, BT3	76.44	[49]
34	3-((6-methylpyrazin-2-yl)methyl)-1H-indole	223.110	224.118	[M+H]^+^	C_14_H_13_N_3_	10.0	0.84	12.09	PC1	84.57	[50]
35	*N*-Lauryldiethanolamine	273.266	274.275	[M+H]^+^	C_16_H_35_NO_2_	0.0	2.34	13.80 (BT2)	BT1, BT2, BT3	99.17	[51]
36	2-*n*-Hexyl-5-*n*-propyl resorcinol	236.177	237.185	[M+H]^+^	C_15_H_24_O_2_	4.0	1.02	18.05 (BT1)	BT1, BT2, BT3	76.54	[52]
37	2-Hexyl-5-methyl resorcinol	208.146	209.154	[M+H]^+^	C_13_H_20_O_2_	4.0	2.33	15.63	BT1	71.60	[53]

Note: C.N (Compound Number), RDBE (Ring Double Bond Equivalents). The isolate name has been given with Retention time for better tracking.

**Table 3:** The list of annotated compounds using GNPS in *Streptomyces* species BT1, BT2, BT3, and PC1.

S.N.	Comp. Name	Accurate Mass (Da)	Precursor Ion	Adduct Type	MS^2^ Fragmentation Pattern	Molecular Formula	Retention Time (min)	Bacterial Source	Error (ppm)	Reference
1	3-*epi*-Xestoaminol C	229.241	230.247	[M+H]^+^	212.237, 66.070, 55.054, 44.050	C_14_H_31_NO	13.85	BT1, BT2, BT3, PC1	4.4	[59]
2	4-Aminobenzoic acid	137.048	138.055	[M+H]^+^	138.049, 120.040, 77.034, 65.035	C_7_H_7_NO_2_	4.85	PC1	0.0	[60]
3	5-Aminovaleric acid	117.079	100.076	[M+H-H_2_O]^+^	100.076, 72.081, 56.050	C_5_H_11_NO_2_	3.14	BT1, BT2, PC1	0.0	[61]
4	(−)-*α*-Bisabolol	222.372	205.195	[M+H-H_2_O]^+^	205.195, 121.100, 93.069, 81.069	C_15_H_26_O	18.84	BT2	0.1	[62]
5	Anthranilic acid	137.048	138.055	[M+H]^+^	120.044, 92.049, 965.038	C_7_H_7_NO_2_	8.43	BT1, BT2, PC1	7.2	[63]
6	Cyclo-[l-(4-hydroxy-pro)-l-leu]	226.132	227.138	[M+H]^+^	199.153, 181.142, 86.078	C_11_H_18_N_2_O_3_	7.37	BT1, BT2, BT3, PC1	8.8	[64]
7	Cyclo(l-leu-trans-4-hydroxy-l-pro)	226.132	227.139	[M+H]^+^	199.153, 181.142, 86.078	C_11_H_18_N_2_O_3_	6.92	BT1, BT2, BT3, PC1	4.3	[65]
8	Cyclo(d-6-Hyp-l-Phe)	260.116	261.124	[M+H]^+^	120.080, 103.054, 86.060	C_14_H_16_N_2_O_3_	8.92	BT1, BT2, BT3,	3.8	[66]
9	Cyclo(l-Phe-d-Pro)	244.121	245.128	[M+H]^+^	154.072, 120.080, 70.065	C_14_H_16_N_2_O_2_	9.40	BT1, BT2, BT3, PC1	4.0	[67]
10	Cyclo(l-Val-l-Pro)	196.121	197.129	[M+H]^+^	124.112, 98.060, 70.066	C_10_H_16_N_2_O_2_	7.03	BT1, BT2, BT3, PC1	0.0	[68]
11	Tryptophol	161.084	162.091	[M+H]^+^	143.072, 130.064, 115.053, 91.054	C_10_H_11_NO	10.29	BT1, BT3, PC1	6.1	[40]
12	Surfactin C1	1035.683	1036.690	[M+H]^+^	1036.687, 699.466, 582.410, 455.286, 356.245	C_53_H_93_N_7_O_13_	22.59	BT1	2.94	[25]
13	Lipopeptide NO	993.636	994.644	[M+H]^+^	994.647, 881.554, 554.371, 441.284	C_50_H_87_N_7_O_13_	20.80	BT1	1.04	[69]
14	Surfactin B	1021.667	1044.660	[M+Na]^+^	931.574, 818.491, 728.496, 657.458, 594.348	C_52_H_91_N_7_O_13_	22.23	BT1	0.9	[70]
15	Lauryldiethanolamine	273.267	274.274	[M+H]^+^	274.274, 256.263, 106.086, 88.075	C_16_H_35_NO_2_	13.78	BT1, BT2, BT3, PC1	7.0	[71]
16	Tetradecyldiethanolamine	301.298	302.305	[M+H]^+^	302.305, 284.294, 106.086, 88.075	C_18_H_39_NO_2_	15.30	BT1, BT2, BT3, PC1	6.6	[72]
17	Indole-3-carbinol	147.068	130.066	[M+H-H_2_O]^+^	103.056, 95.050, 77.038	C_9_H_9_NO	9.43	BT1, BT2, BT3, PC1	7.6	[73]
18	Cyclo(leucylprolyl)	210.137	211.144	[M+H]^+^	211.145	C_11_H_18_N_2_O_2_	9.48	BT1, BT2, BT3, PC1	4.7	[26]
19	*N*-acetyl-2-phenylethylamine	163.100	164.107	[M+H]^+^	105.070	C_10_H_13_NO	9.78	BT1, BT2, BT3, PC1	6.1	[74]
20	*N*-acetyltyramine	179.095	180.102	[M+H]^+^	121.065, 103.054, 93.070	C_10_H_13_NO_2_	7.19	BT1, BT2, BT3, PC1	5.5	[35]
21	N6-(delta2-isopentenyl)adenine	203.117	204.124	[M+H]^+^	148.063, 136.063, 69.071	C_10_H_13_N_5_	8.10	BT1, BT3, PC1	9.86	[75]
22	*N*-[2-(1*H*-indol-3-yl)ethyl]acetamide	202.111	203.118	[M+H]^+^	144.081	C_12_H_14_N_2_O	10.21	BT1, BT2	0.0	[76]
23	Brevianamide F	283.132	284.139	[M+H]^+^	130.064	C_16_H_17_N_3_O_2_	9.19	BT1, BT2, BT3, PC1	6.9	[73]
24	Normetanephrine	183.090	184.097	[M+H]^+^	134.059, 106.065, 91.054, 77.038	C_9_H_13_NO_3_	2.41	BT1, BT2, BT3, PC1	0.0	[77]
25	13-Docosenamide	337.334	338.342	[M+H]^+^	149.132, 121.101, 97.101	C_22_H_43_NO	22.36	BT1, BT2, BT3, PC1	0.0	[78]

## Data Availability

Data available on request from the corresponding author.

## Supplementary Materials

The following supporting information can be downloaded at: https://www.mdpi.com/article/10.3390/ijms25084193/s1.
