# Supplementary material for: Metabolic Comparison and Molecular Networking of Antimicrobials in Streptomyces Species
Source: Int J Mol Sci. 2024 Apr 10;25(8):4193. doi: 10.3390/ijms25084193 (PMC11050201; doi:10.3390/ijms25084193)
Supplement: Supplementary file 1 [file ijms-25-04193-s001.zip › ijms-2896745-supplementary.pdf]

Metabolic Comparison and Molecular Networking of Antimicrobials in *Streptomyces* Species

Bijaya Bahadur Thapa [1,†], Chen Huo [2,†], Rabin Budhathoki [1], Pratiksha Chaudhary [1], Soniya Joshi [1], Purna Bahadur Poudel [3], Rubin Thapa Magar [3], Niranjan Parajuli [1,3], Ki Hyun Kim [2,*] and Jae Kyung Sohng [3,*]

[1] Central Department of Chemistry, Tribhuvan University, Kirtipur 44618, Kathmandu, Nepal; bijaya.chem@gmail.com (B.B.T.); rabin.bc.992@gmail.com (R.B.); pratikshachaudhary274@gmail.com (P.C.); soniyajoshi157@gmail.com (S.J.); niranjan.parajuli@cdc.tu.edu.np (N.P.)
[2] School of Pharmacy, Sungkyunkwan University, Suwon 16419, Republic of Korea; huochen_0213@163.com
[3] Institute of Biomolecule Reconstruction (iBR), Department of Life Science and Biochemical Engineering, Sun Moon University, Asan 31460, Republic of Korea; pbspoudel@gmail.com (P.B.P.); magarrubin@gmail.com (R.T.M.)
* Correspondence: khkim83@skku.edu (K.H.K.); sohng@sunmoon.ac.kr (J.K.S.)
† These authors contributed equally to this study.

Table S1. List of soil samples collected from different regions of Nepal.

Samples	Locations	Altitude	Coordinates	Source of Soils
BT1	Low Camp (Kaski)	2680 m	28.2622° N, 84.0167° E	Forest
BT2	Jomsom city (Mustang)	2743 m	28.9985° N, 83.8473° E	Agricultural Field
BT3	Baglung	1010 m	28.3641° N, 83.2078° E	Hilly Area
PC1	Khaptad National Park	2500 m	29°16′12″N, 80°59′24″E	Forest

Table S2. Zone of inhibition of various bacteria EA extracts against tested pathogenic bacteria.

Actinomycetes	*Staphylococcus aureus* (mm)	*Escherichia coli* (mm)	*Salmonella typhi* (mm)	*Shigella sonnei* (mm)	*Klebsiella pneumoniae* (mm)
BT1	18	14	-	25	-
BT2	16	20	-	23	-
BT3	15	16	12	20	-
PC1	31	32	30	30	30
Neomycin	28	25	25	25	23

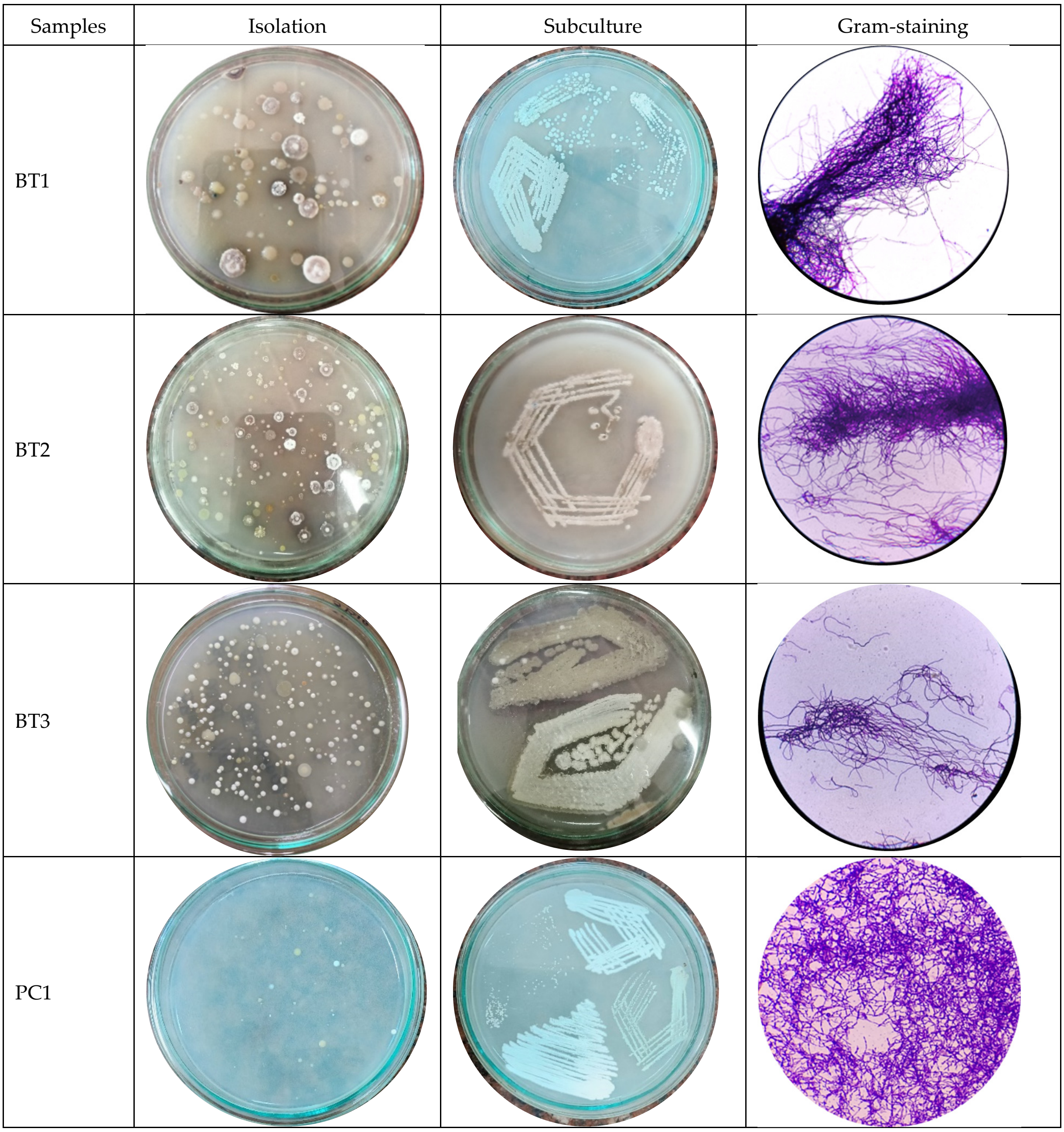

Figure S1. (A) Isolation of four soil samples by using ISP4 media after one week of incubation at 28 °C which represents the morphology of colonies (white or greyish white), (B) subculture of those isolates by picking up the immersed colony for pure culture on same media, (C) respective microscopic feature of mycelia at 100X oil immersion of four representative genera of actinomycetes isolates (BT1, BT2, BT3, and PC1).

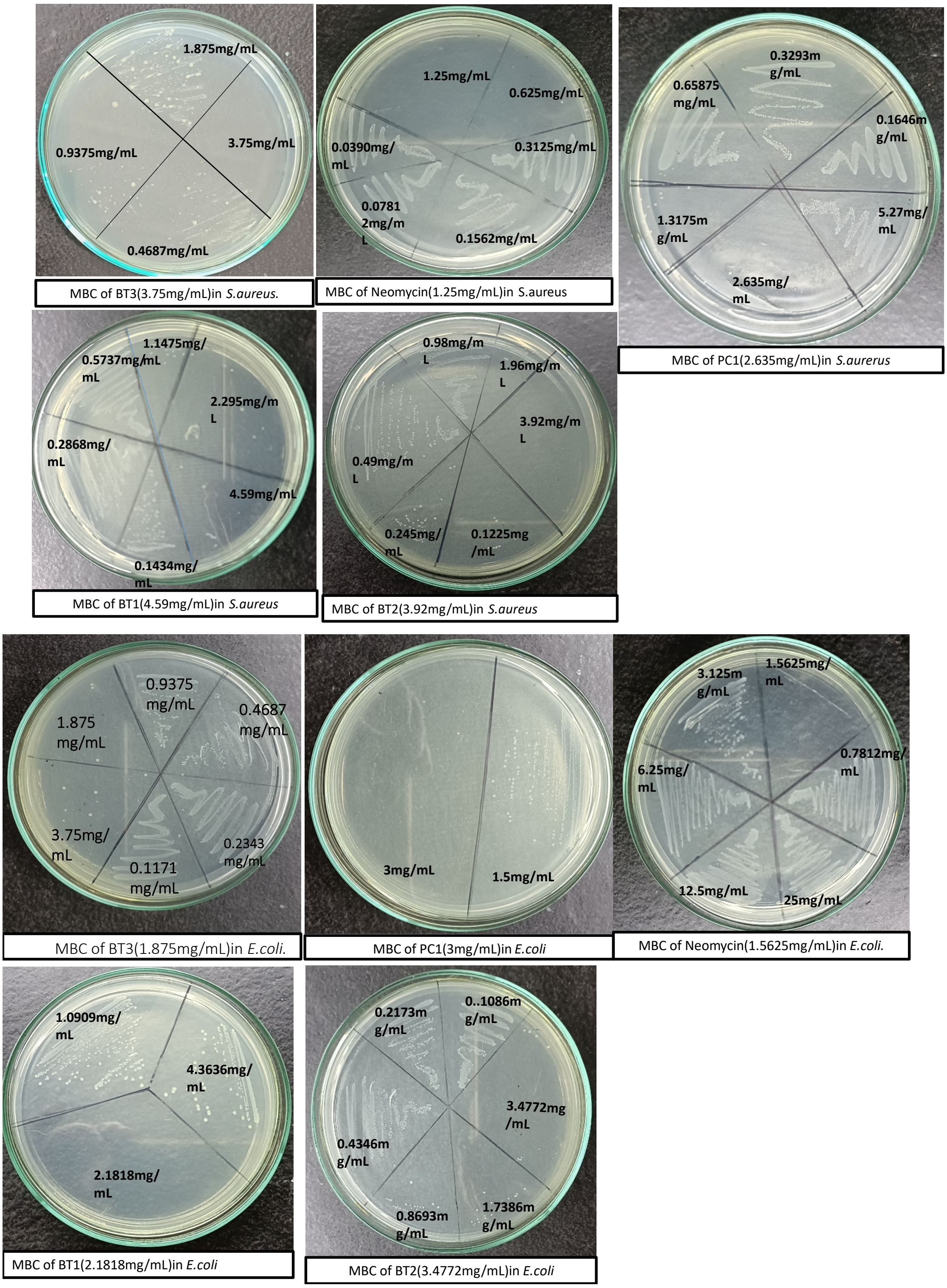

Figure S2. MBC of EA extracts of *Streptomyces* species.

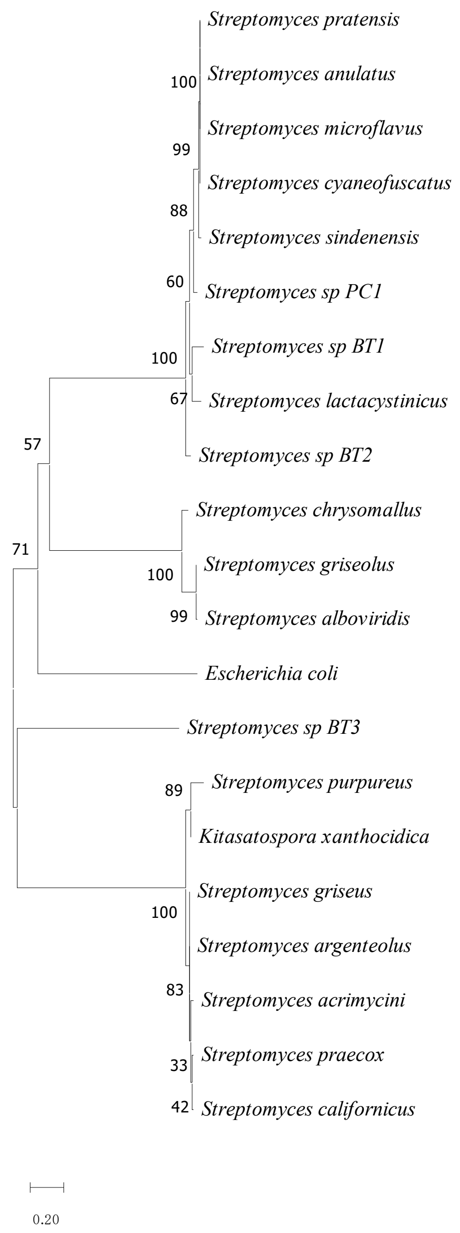

Figure S3. Neighbor-joining phylogenetic tree of 16S rRNA of isolates (*Streptomyces* sp. BT1, BT2, BT3, and PC1). Horizontal branch lengths proportional to the estimated number of nucleotide substitutions, and bootstrap probabilities (as percentages), as determined for 1000 resamplings, are given above and beside the internal branches. The bar in the lower-left corner indicates 0.20 substitutions per nucleotide position. *Escherichia coli* (J01859.1) was used as an outgroup to root the tree.

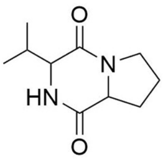

Cyclo(Pro-Val) (**18**)
Exact Mass: 196.121
$C_{10}H_{16}N_2O_2$

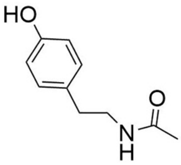

N-acetyltyramine **(19)**
Exact Mass: 179.095
$C_{10}H_{13}NO_2$

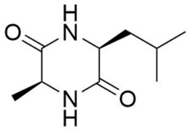

Cyclo(L-Ala-L-Leu) **(20)**
Exact Mass: 184.121
$C_9H_{16}N_2O_2$

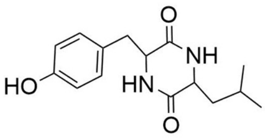

Cyclo(Tyr-Leu) **(21)**
Exact Mass: 276.147
$C_{15}H_{20}N_2O_3$

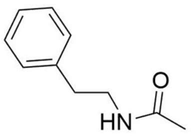

N-Phenethylacetamide (**25**)
Exact Mass: 163.10
$C_{10}H_{13}NO$

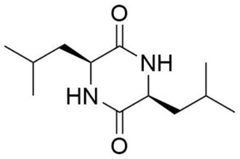

Cyclo(L-leucyl-L-leucyl) (**26**)
Exact Mass: 226.17
$C_{12}H_{22}N_2O_2$

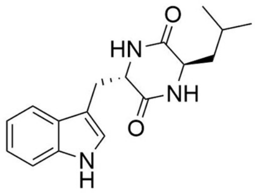

Cyclo-(D-Leu-L-Trp) (**27**)
Exact Mass: 299.16
$C_{17}H_{21}N_3O_2$

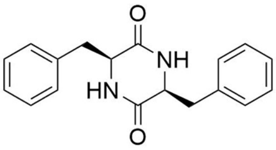

Cyclo(phenylalanyl-phenylalanyl) (**28**) Exact Mass: 294.14
$C_{18}H_{18}N_2O_2$

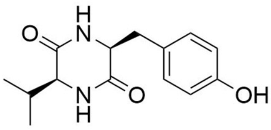

Cyclo(Tyr-Val) (**29**)
Exact Mass: 262.13
$C_{14}H_{18}N_2O_3$

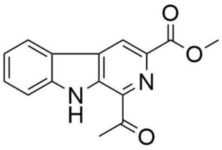

1-Acetyl-3-methoxy-carbonyl-beta-carboline (**30**)
Exact Mass: 268.08
$C_{15}H_{12}N_2O_3$

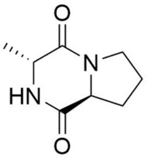

Cyclo(D-Ala-L-Pro) (**31**)
Exact Mass: 168.09
$C_8H_{12}N_2O_2$

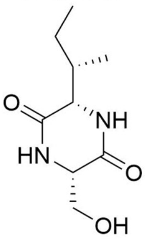

Cyclo(Ile-Ser) (**32**)
Exact Mass: 200.12
$C_9H_{16}N_2O_3$

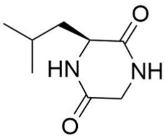

(S)-3-Isobutylpiperazine-2,5-dione (**33**)
Exact Mass: 170.11
$C_8H_{14}N_2O_2$

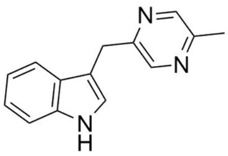

3-[(6-Methylpyrazin-2-yl)methyl]-1H-indole (**34**)
Exact Mass: 223.11
$C_{14}H_{13}N_3$

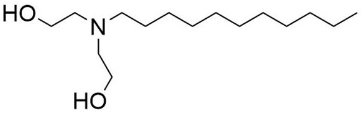

N-Lauryldiethanolamine (**35**)
Exact Mass: 259.25
$C_{15}H_{33}NO_2$

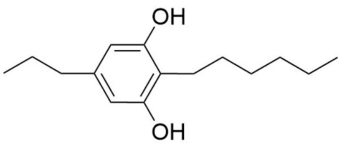

2-n-Hexyl-5-n-propylresorcinol (**36**)
Exact Mass: 236.18
$C_{15}H_{24}O_2$

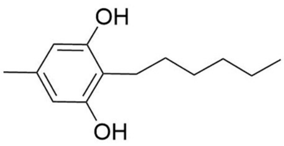

2-Hexyl-5-methylresorcinol (**37**)
Exact Mass: 208.15
$C_{13}H_{20}O_2$

Figure S4. Common annotated compounds in EA extracts of *Streptomyces* sp. PC1, BT1, BT2, and BT3.

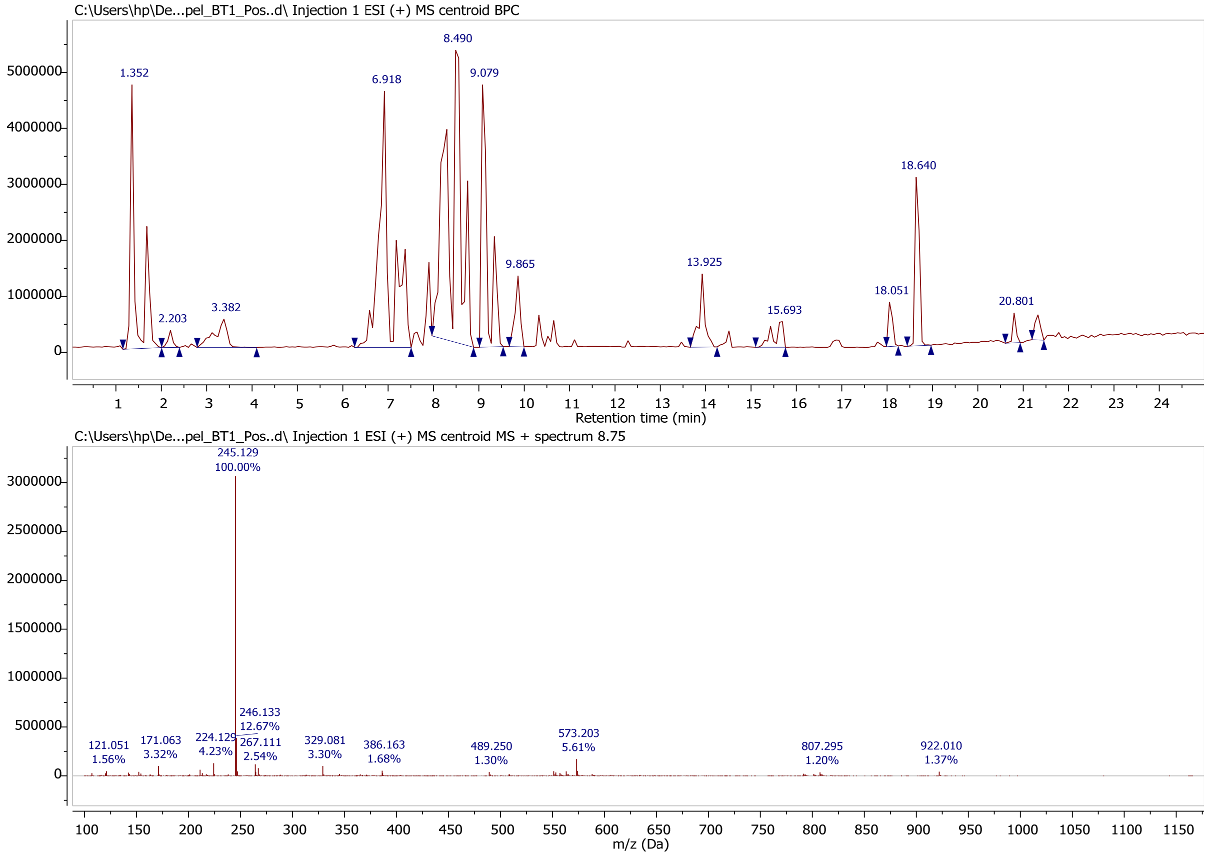

Figure S5. BPC and MS profile of cyclo-(D-pro-D-phe) (**1**)

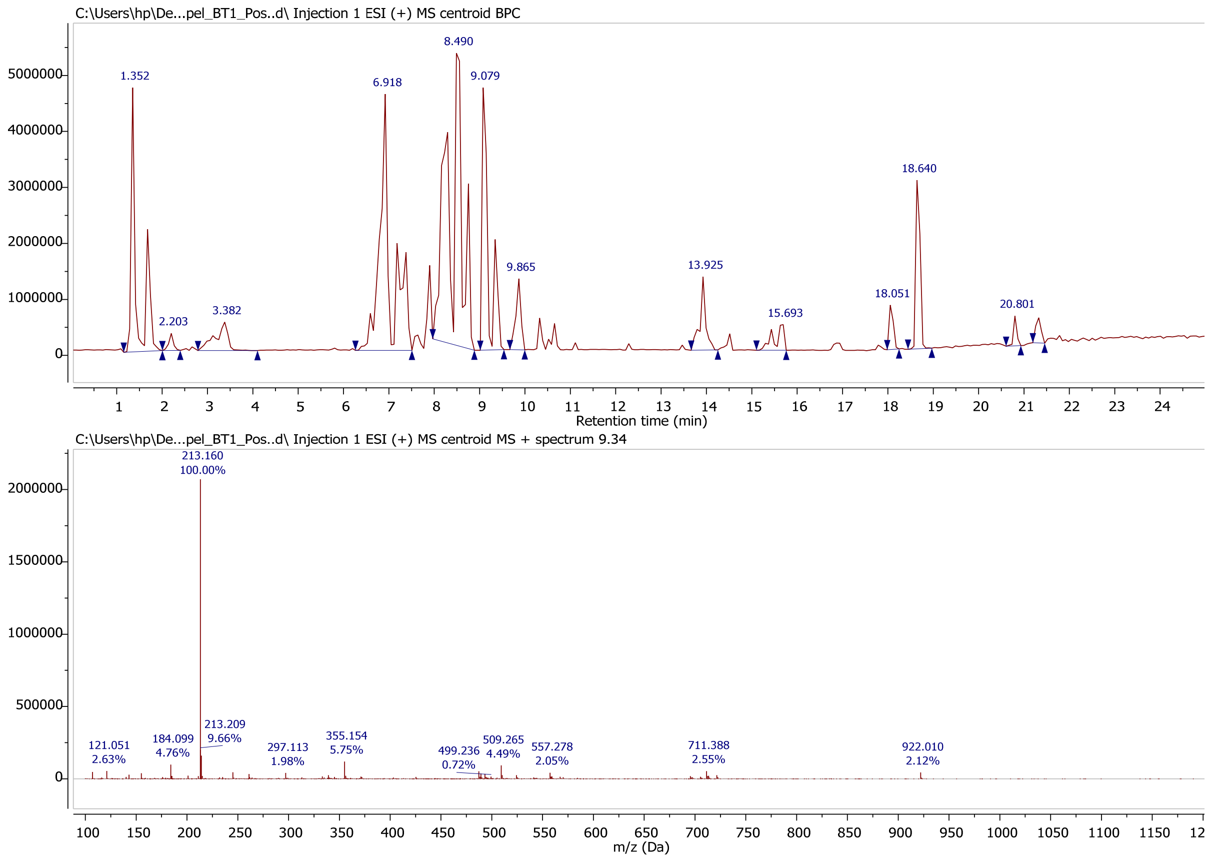

Figure S6. BPC and MS profile of cyclo-(L-val-L-leu) (**2**)

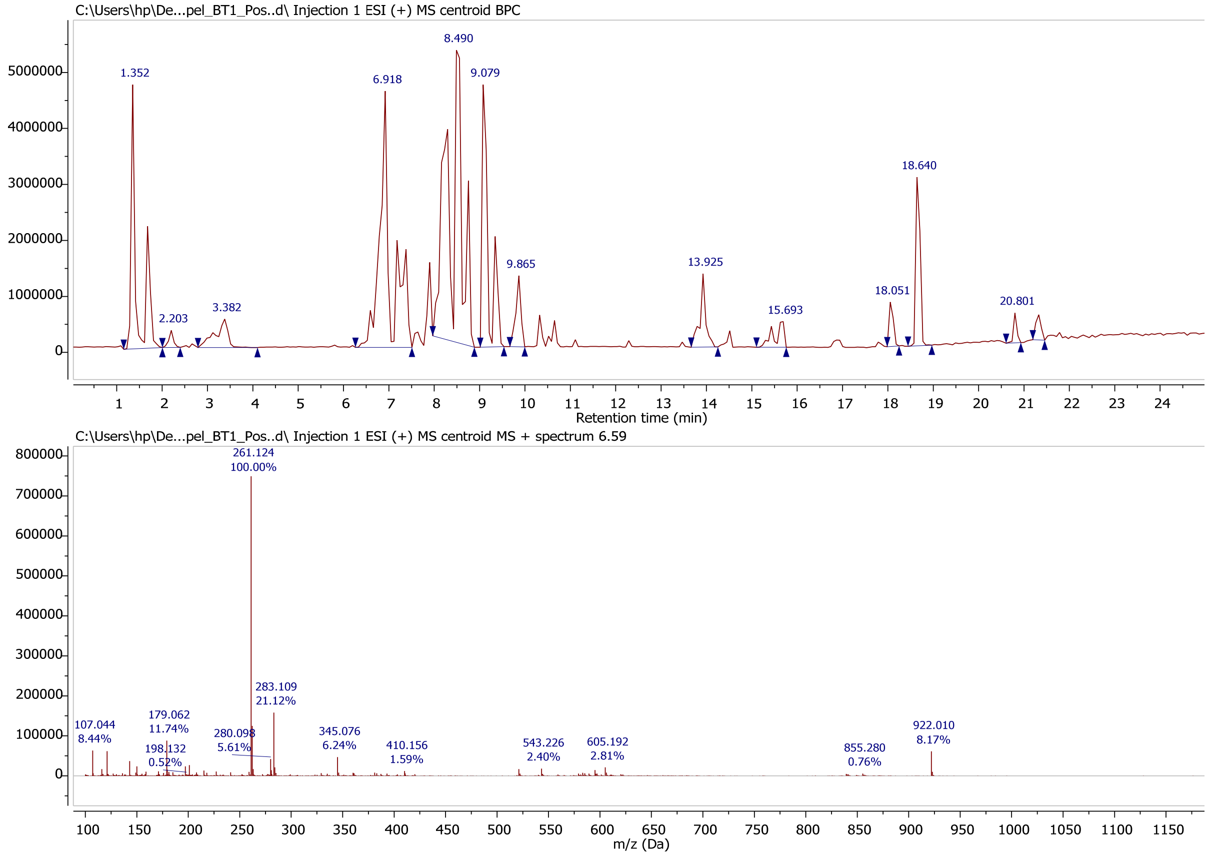

Figure S7. BPC and MS profile of maculosin (**3**)

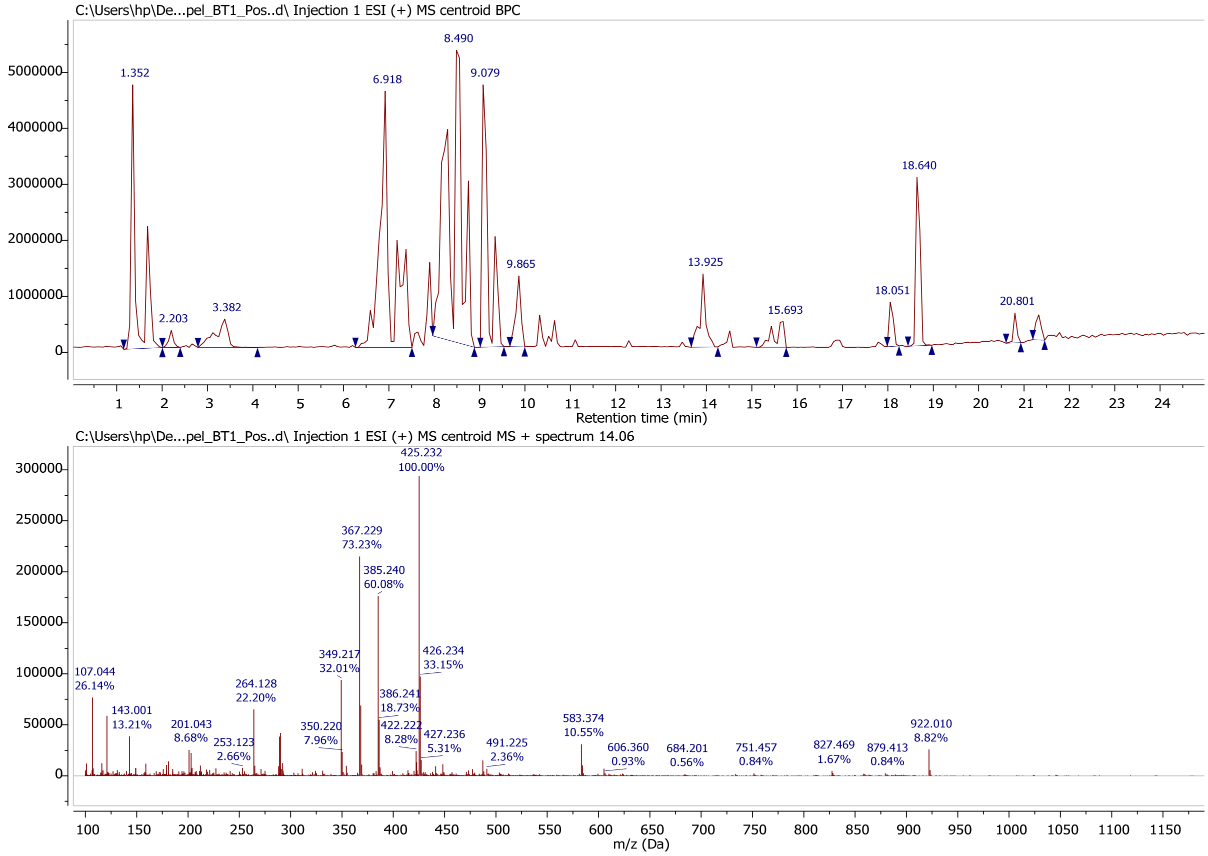

Figure S8. BPC and MS profile of neomarinone (**4**)

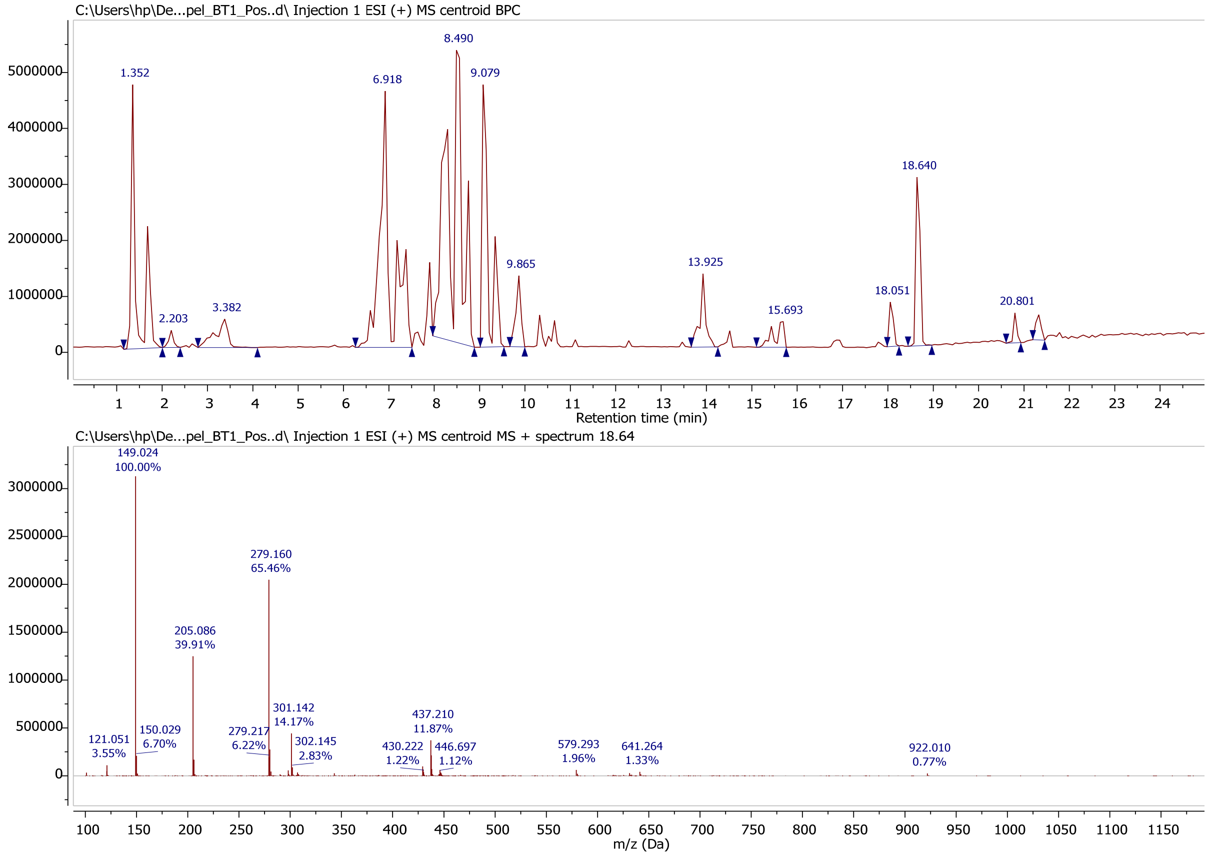

Figure S9. BPC and MS profile of dibutyl phthalate (**5**)

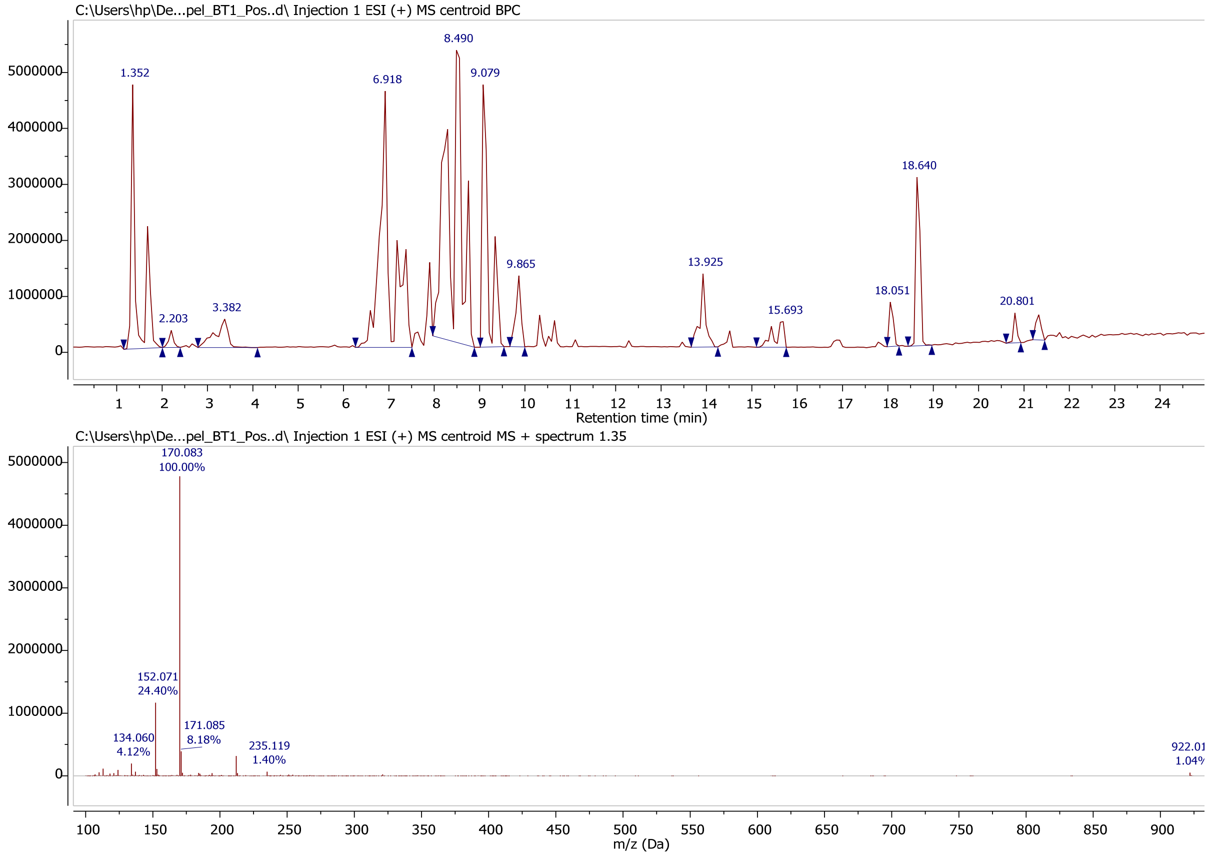

Figure S10. BPC and MS profile of pyridoxine (**6**)

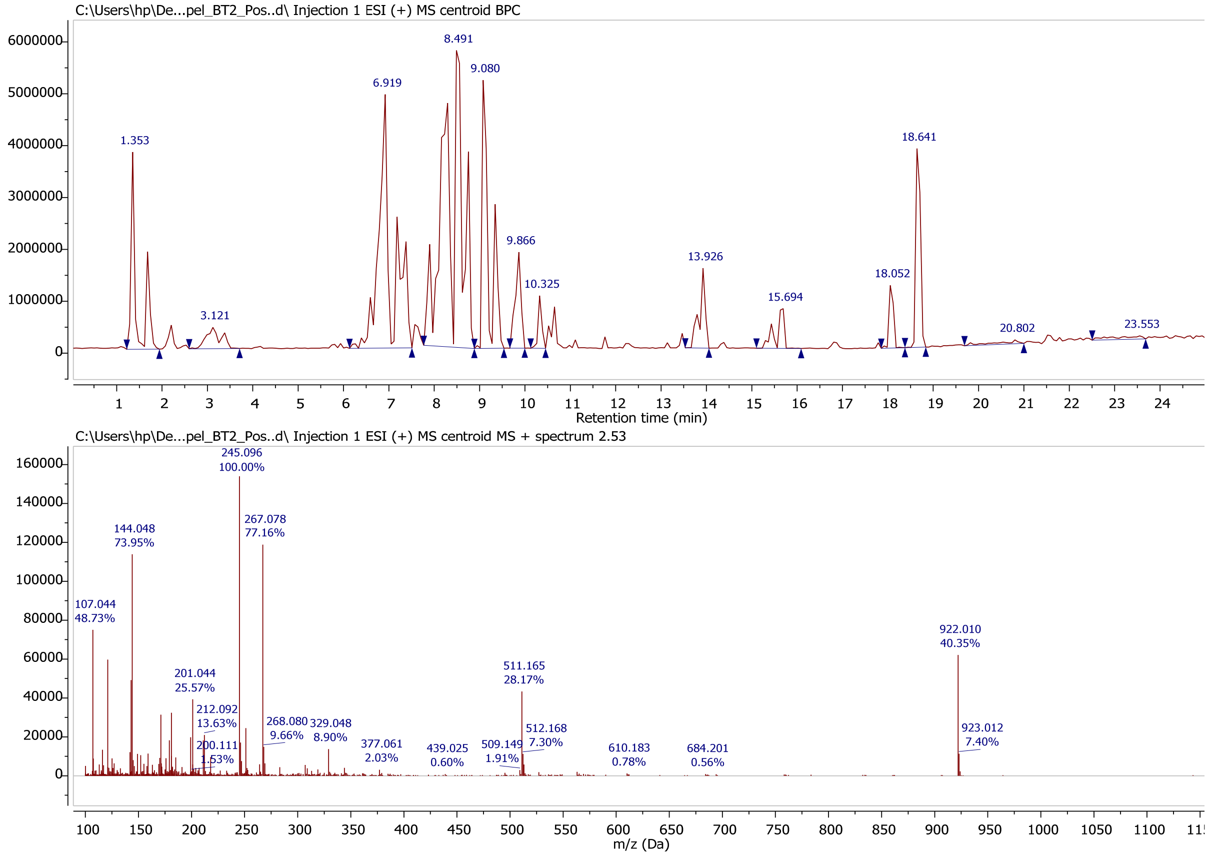

Figure S11. BPC and MS profile of cyclo(L-Pro-L-OMet) (**7**)

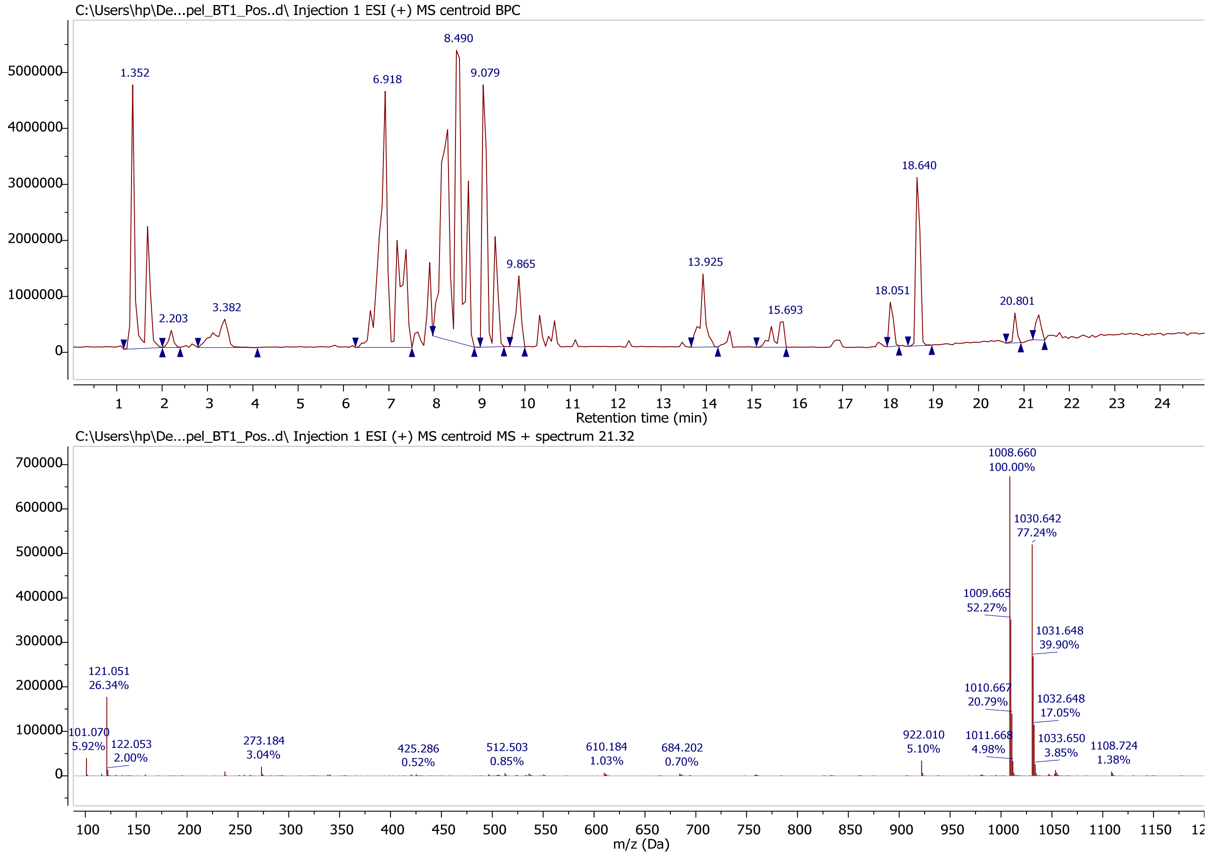

Figure S12. BPC and MS profile of surfactin C13 (**8**)

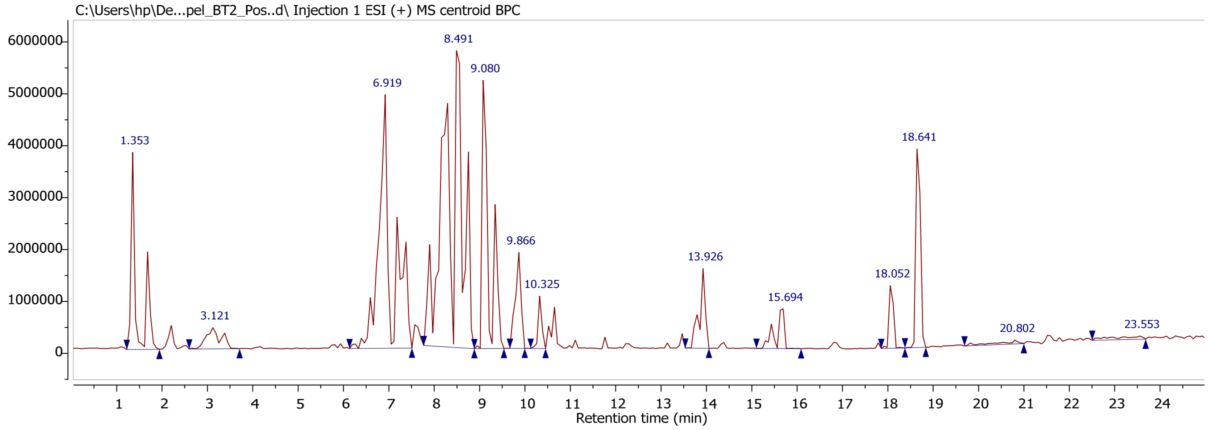

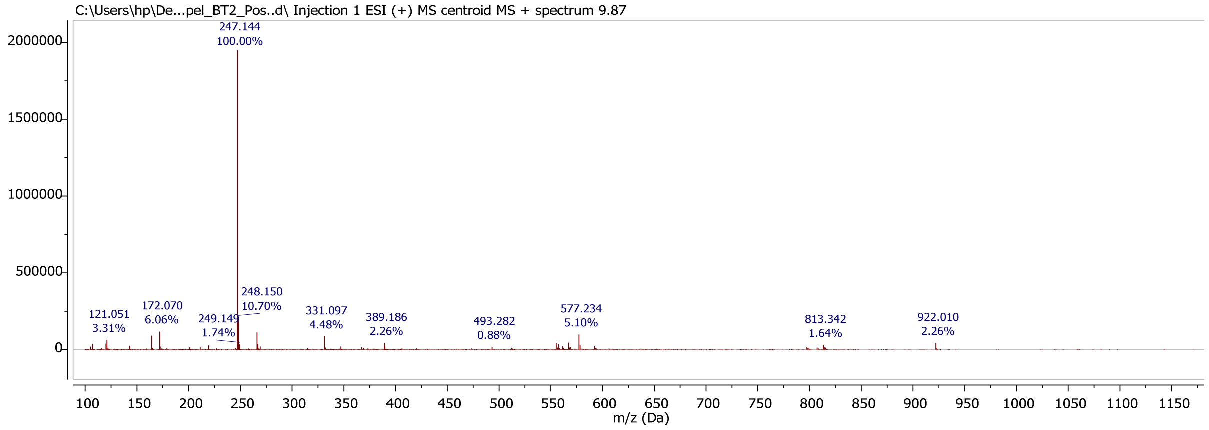

Figure S13. BPC and MS profile of cyclo-(L-valyl-L-phenyl alanyl) (**9**)

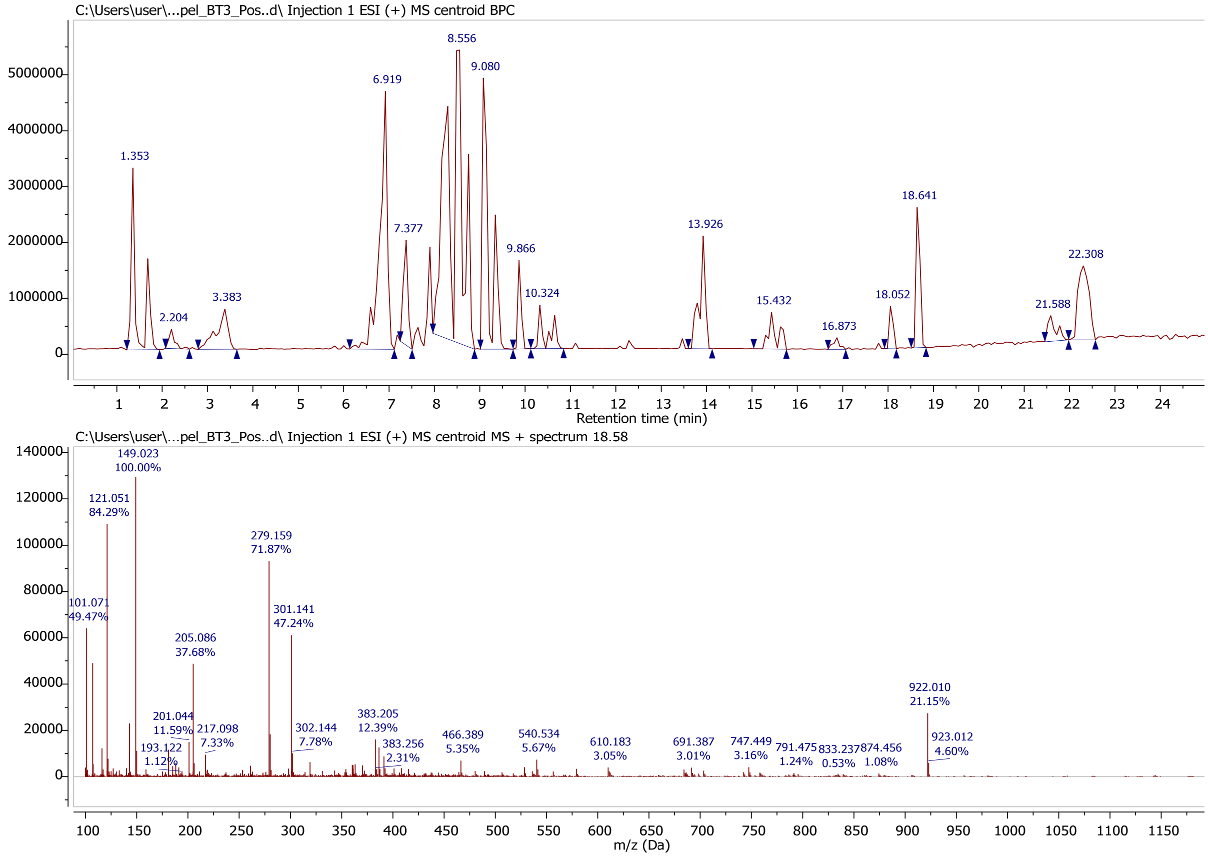

Figure S14. BPC and MS profile of di-n-butyl terephthalate (**10**)

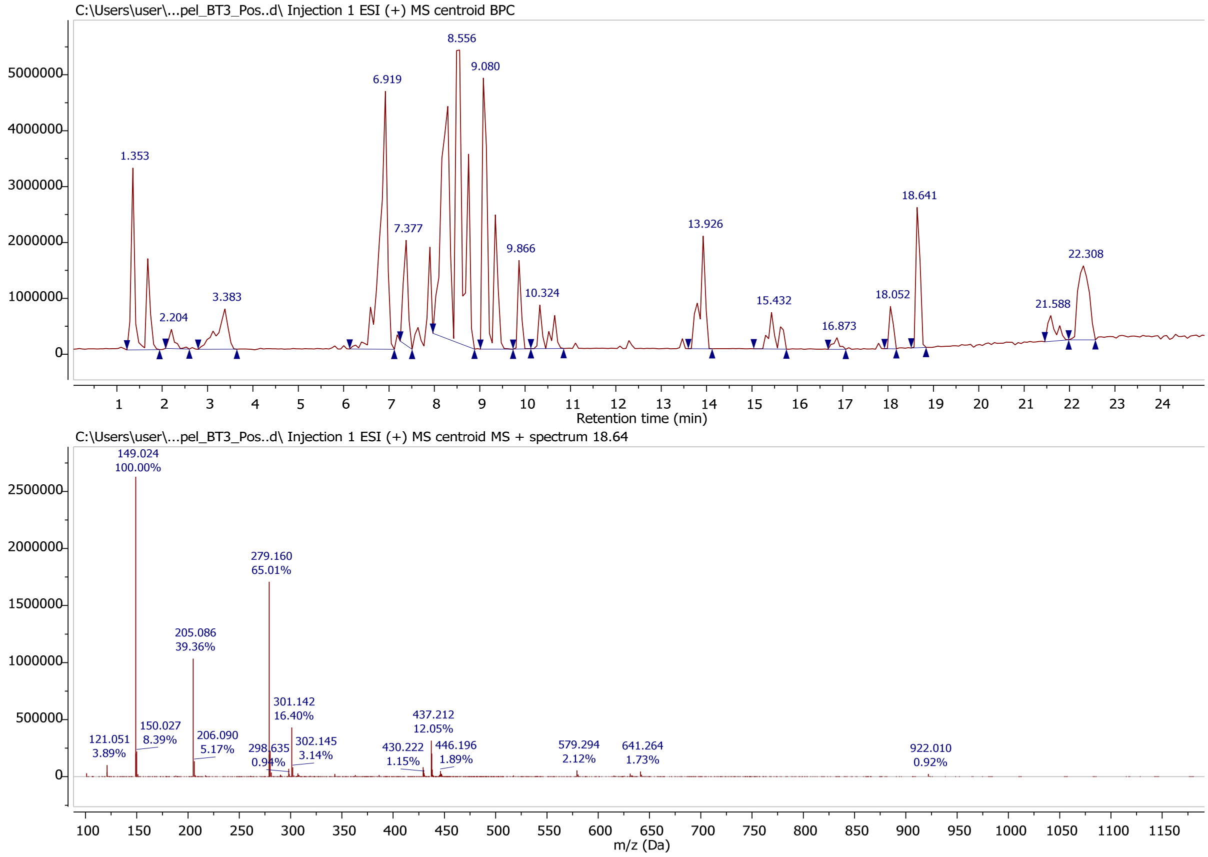

Figure S15. BPC and MS profile of phthalic anhydride (**11**)

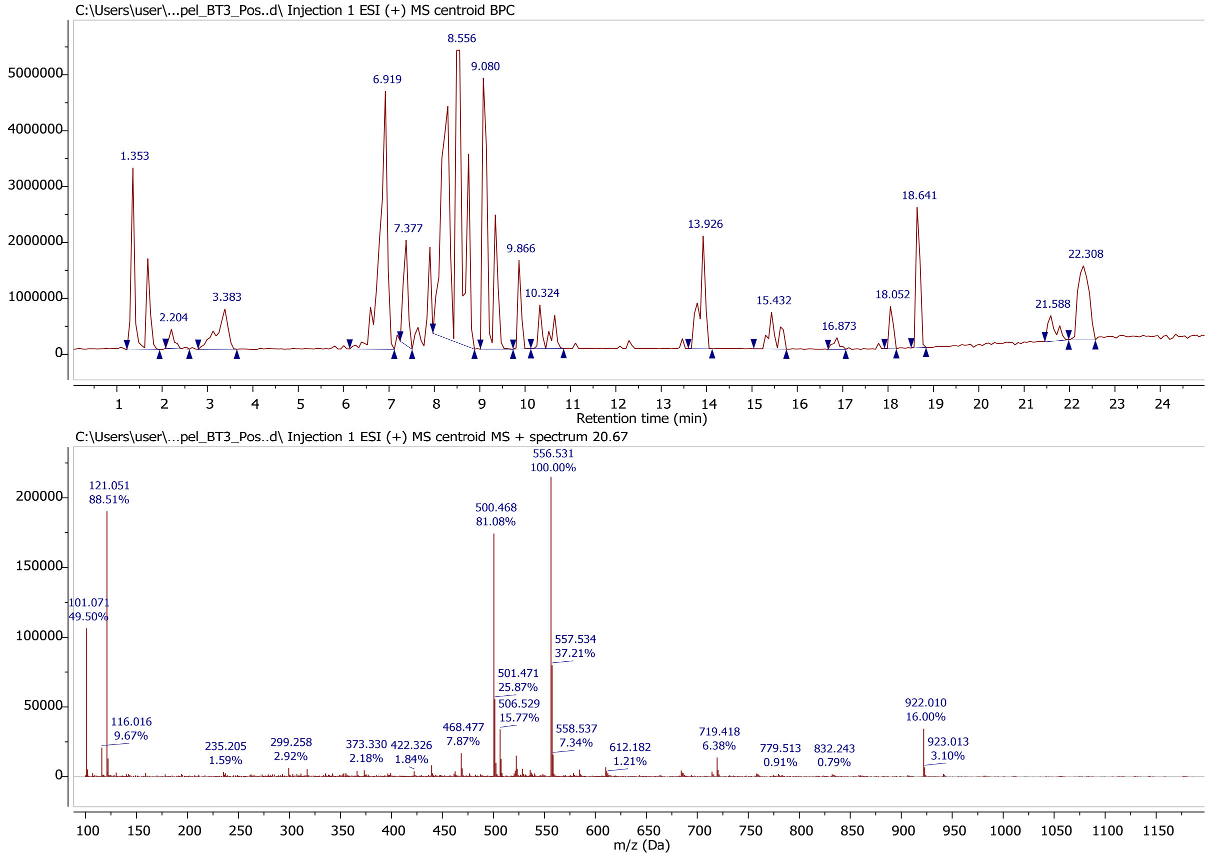

Figure S16. BPC and MS profile of phytoceramide (**12**)

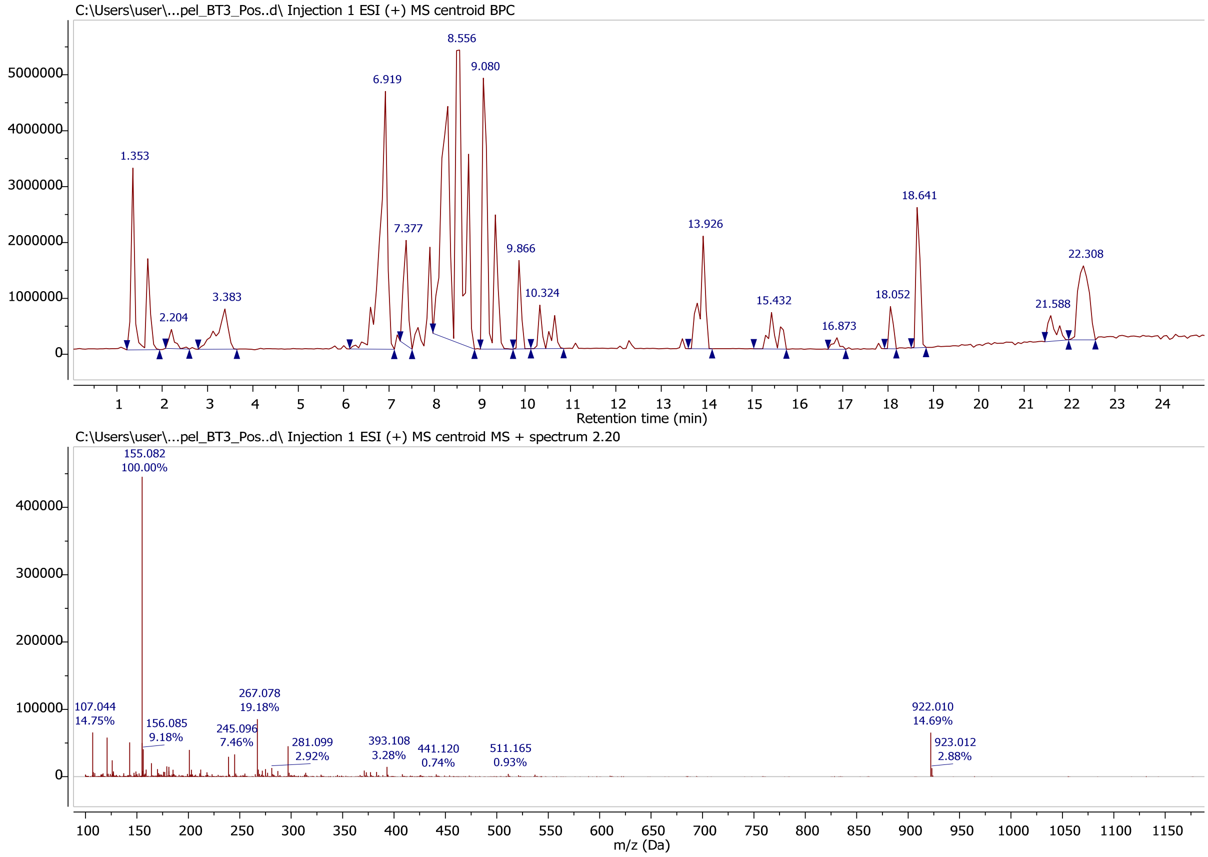

Figure S17. BPC and MS profile of cyclo-(Pro-Gly) (**13**)

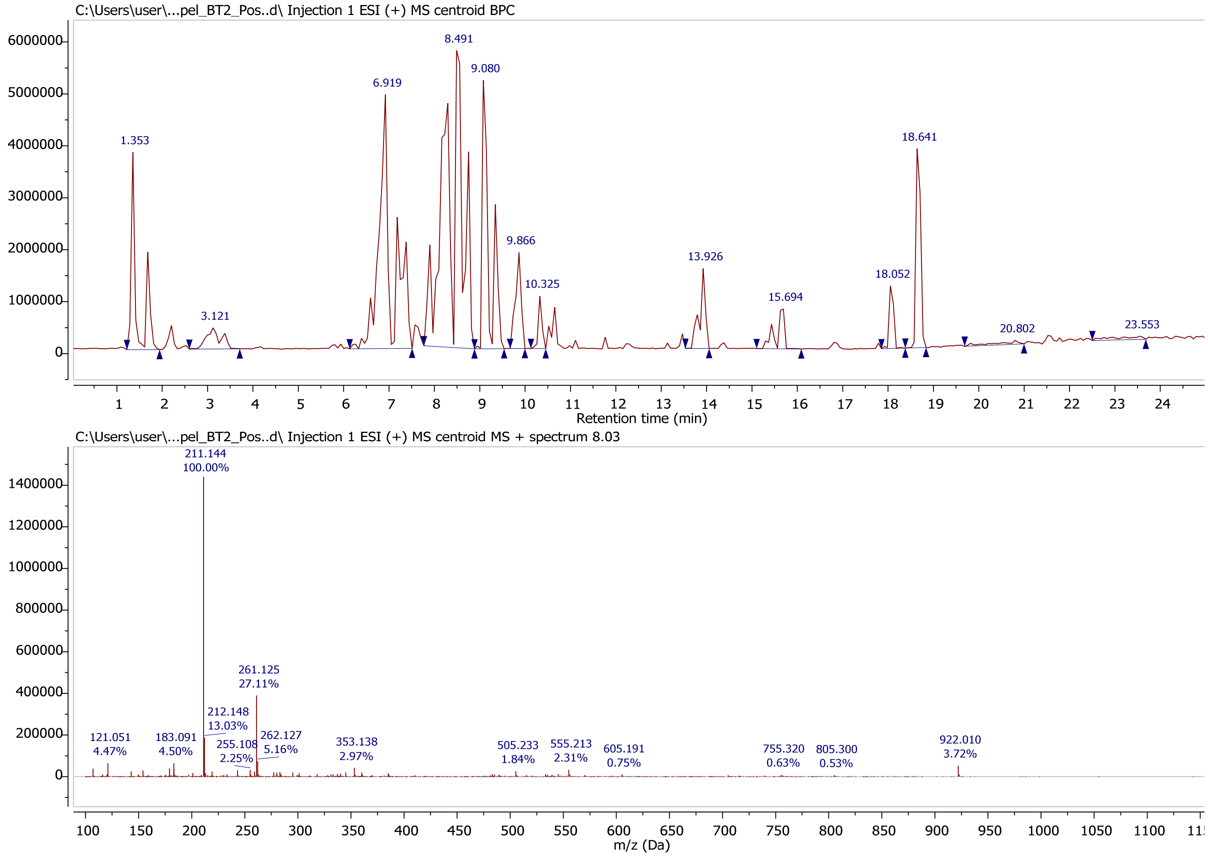

Figure S18. BPC and MS profile of cyclo-(L-leu-L-pro) (**14**)

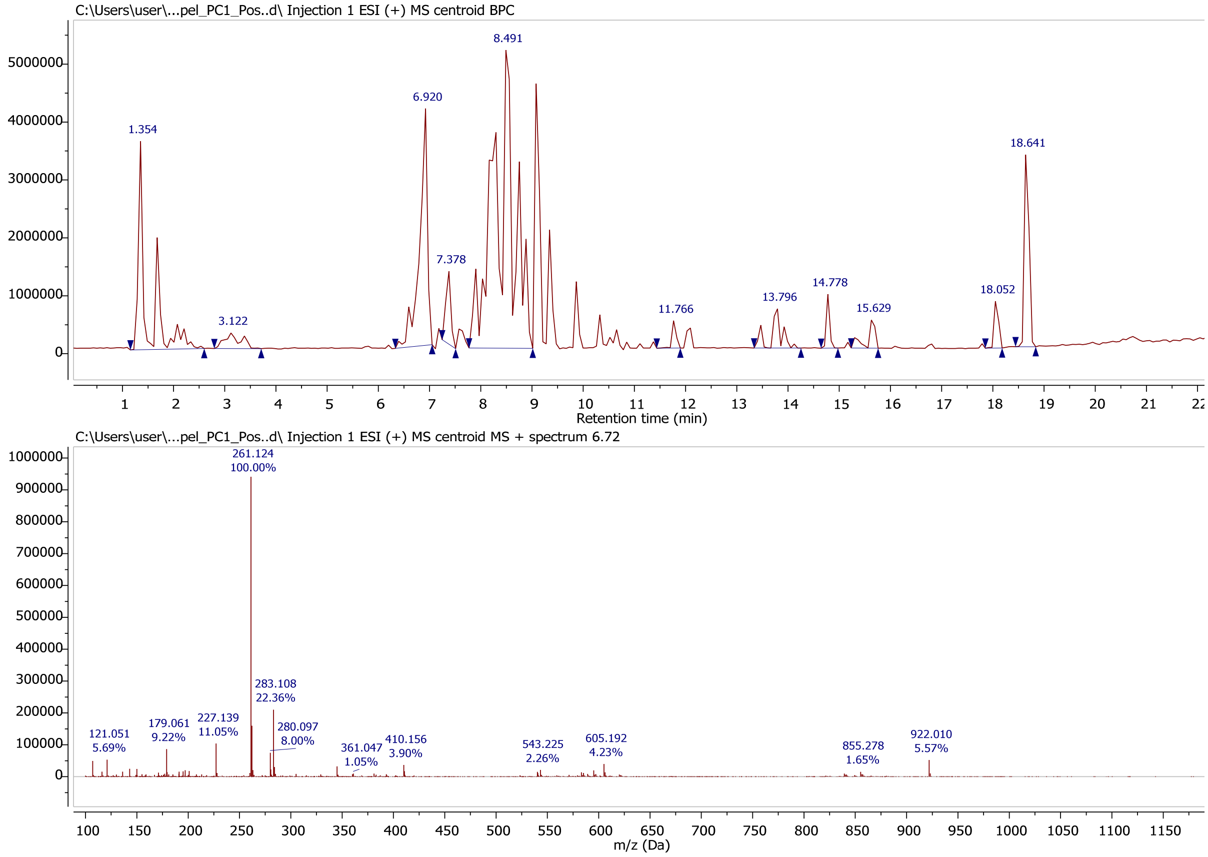

Figure S19. BPC and MS profile of cyclo-(L-phenylalanyl-trans-4-hydroxy-L-proline)(**15**)

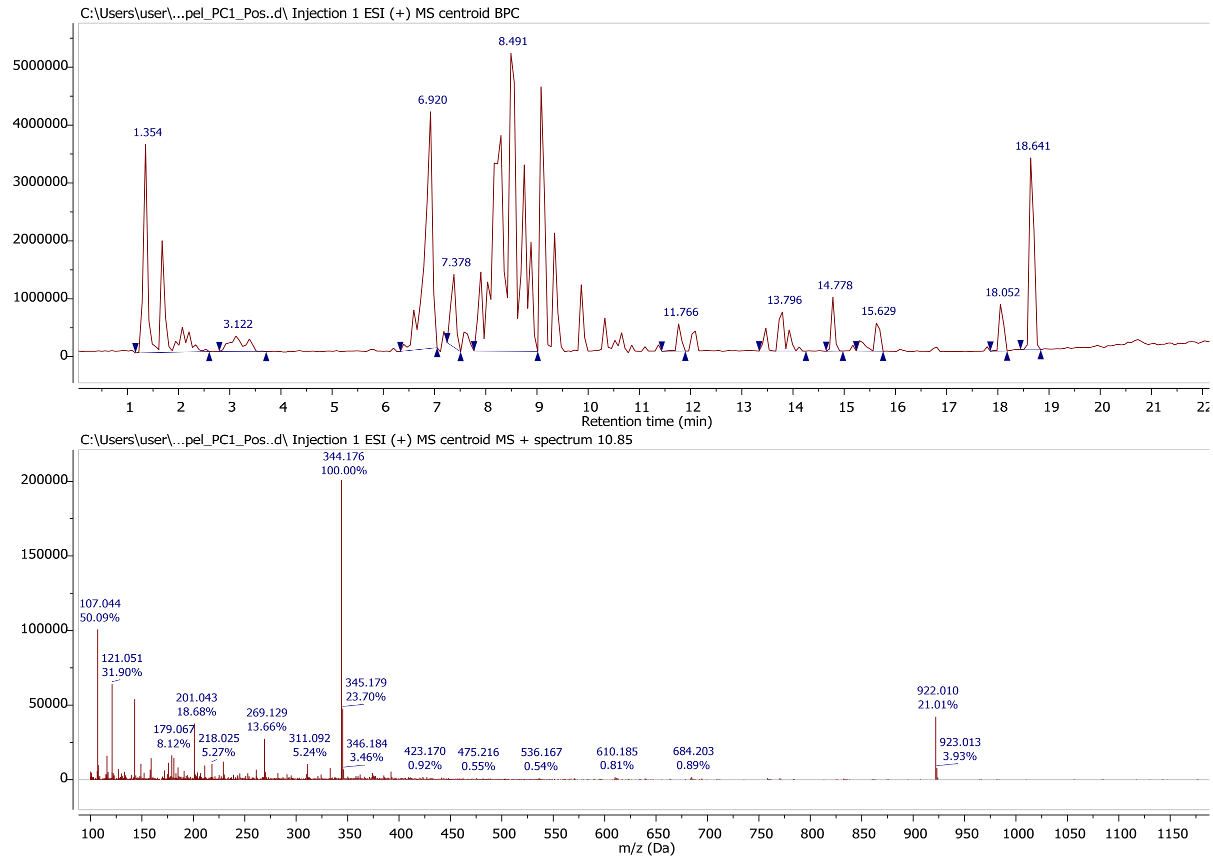

Figure S20. BPC and MS profile of coronafacoyl-L-isoleucine (**16**)

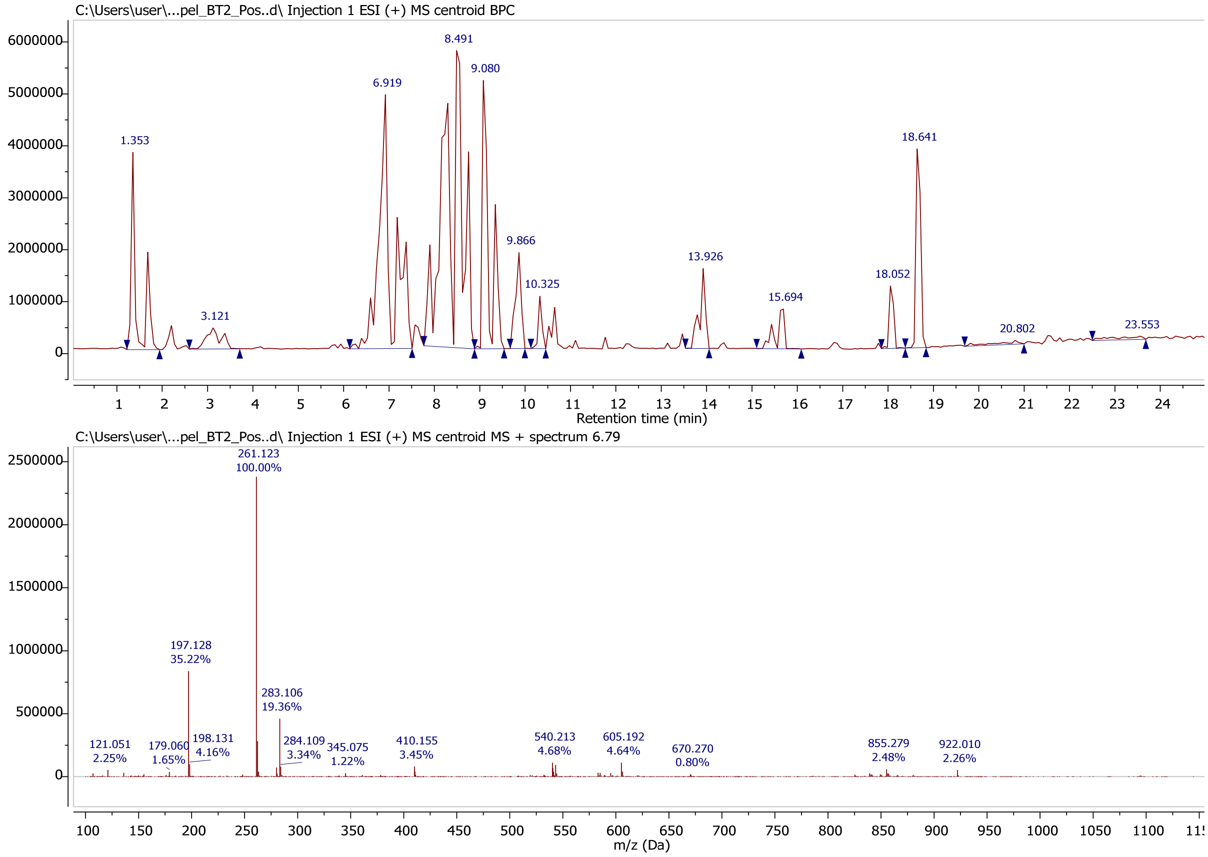

Figure S21. BPC and MS profile of cyclo-(D-Pro-L-Tyr) (**17**)

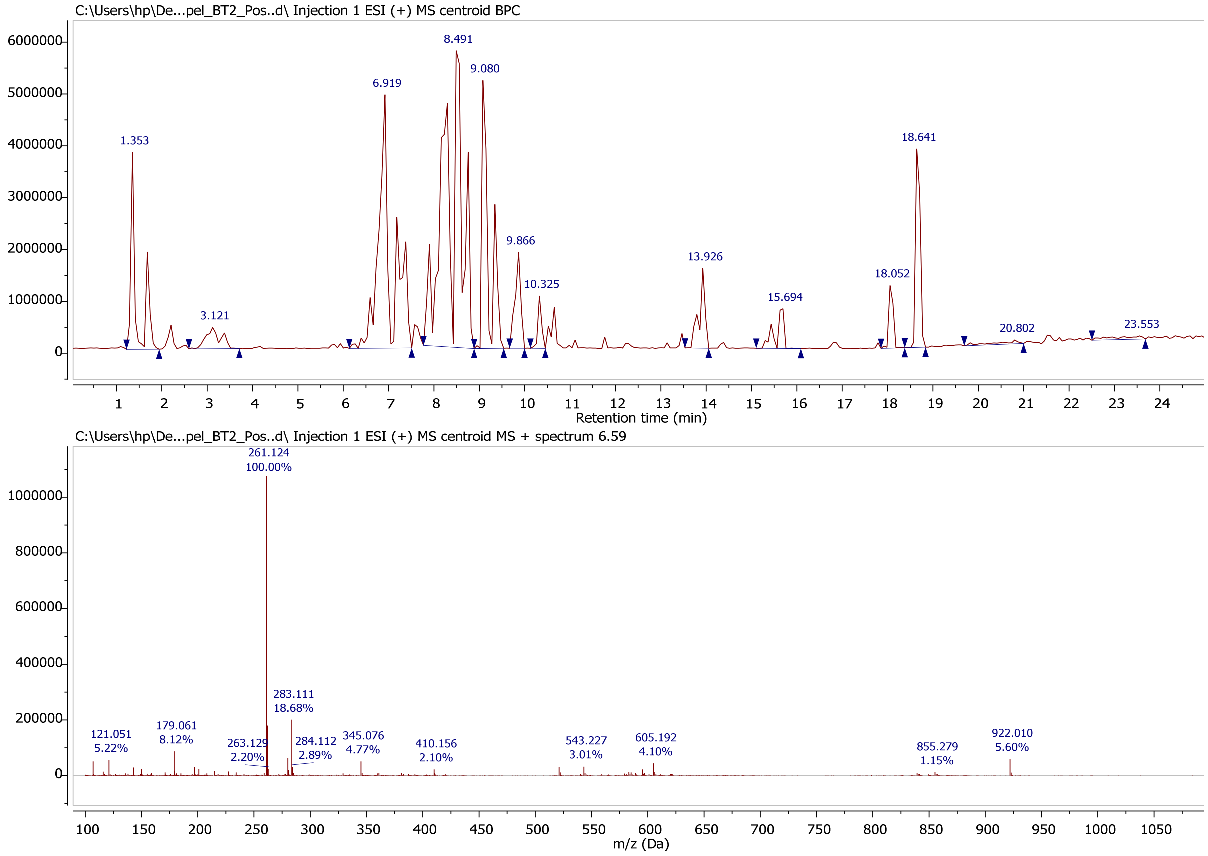

Figure S22. BPC and MS profile of cyclo-(Pro-Val) (**18**)

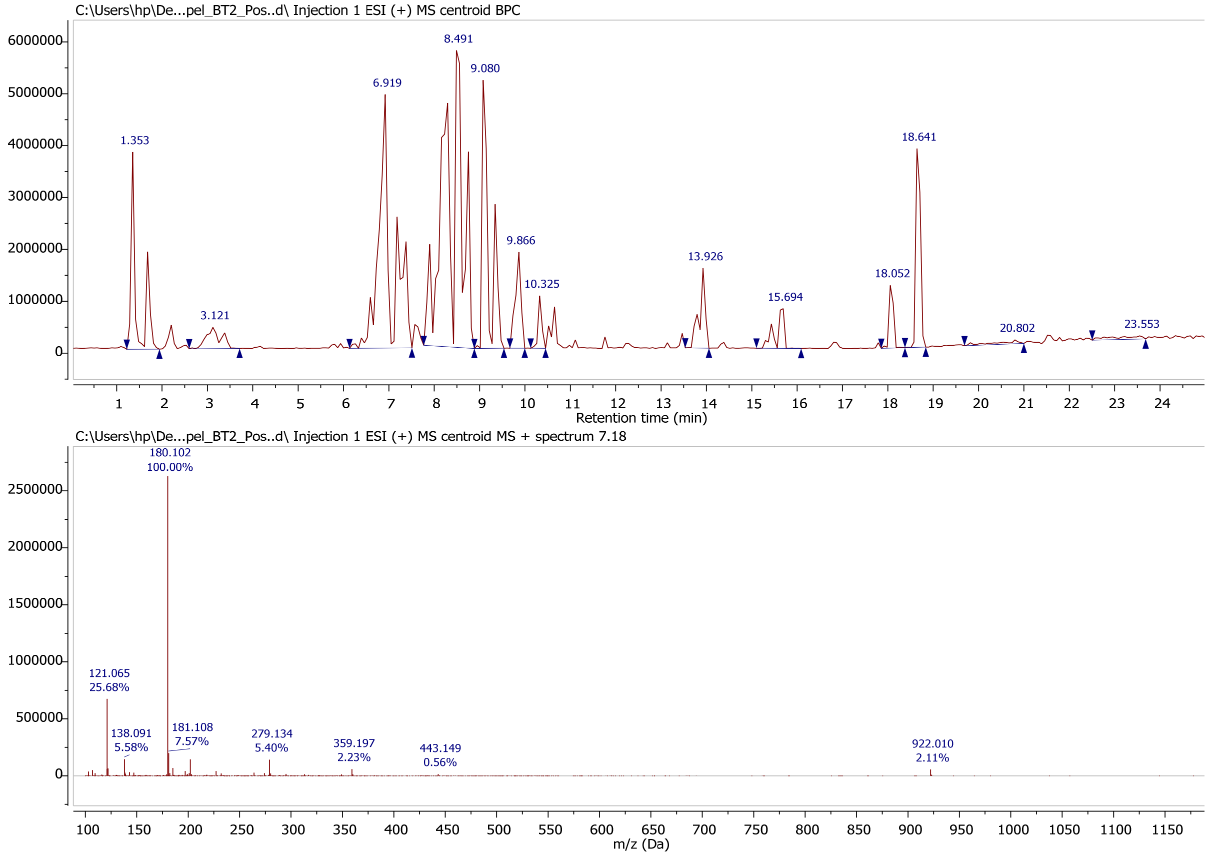

Figure S23. BPC and MS profile of N-acetyltyramine (**19**)

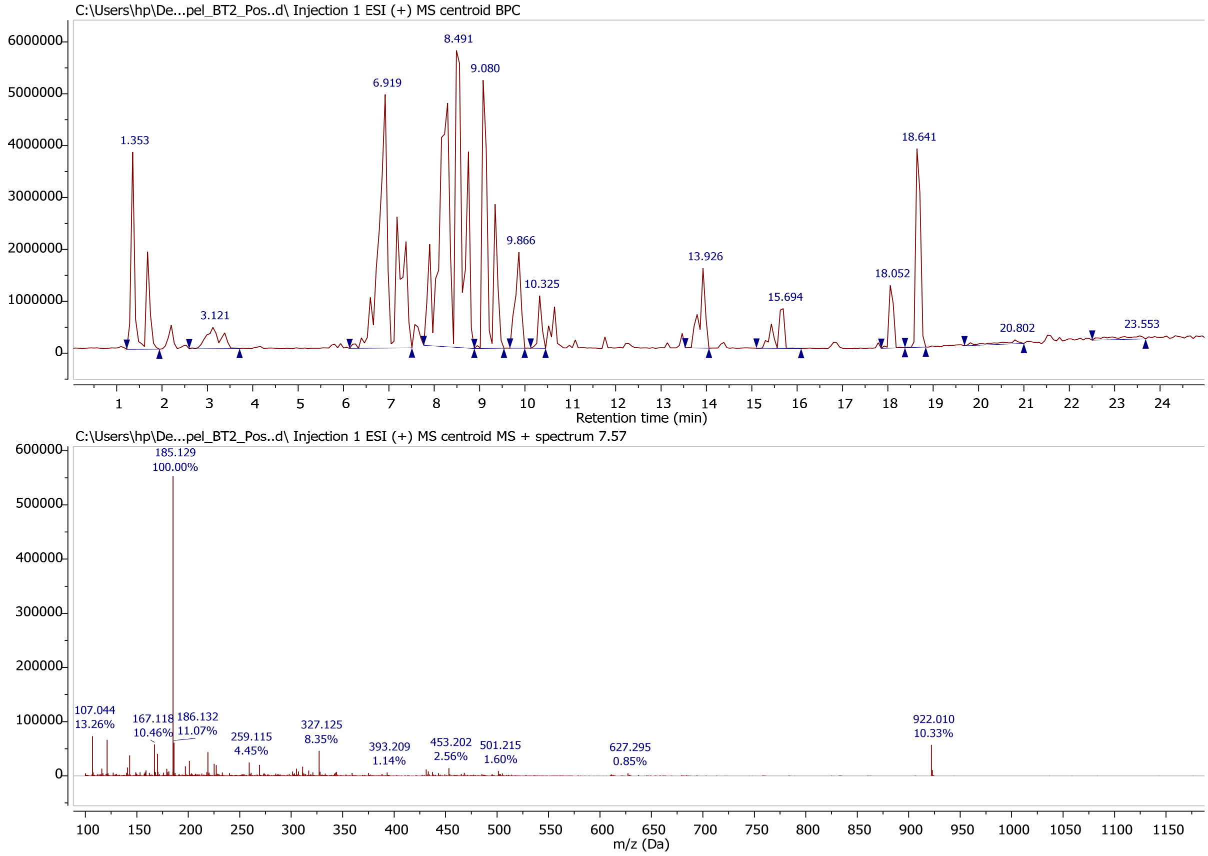

Figure S24. BPC and MS profile of cyclo-(L-Ala-L-Leu) (**20**)

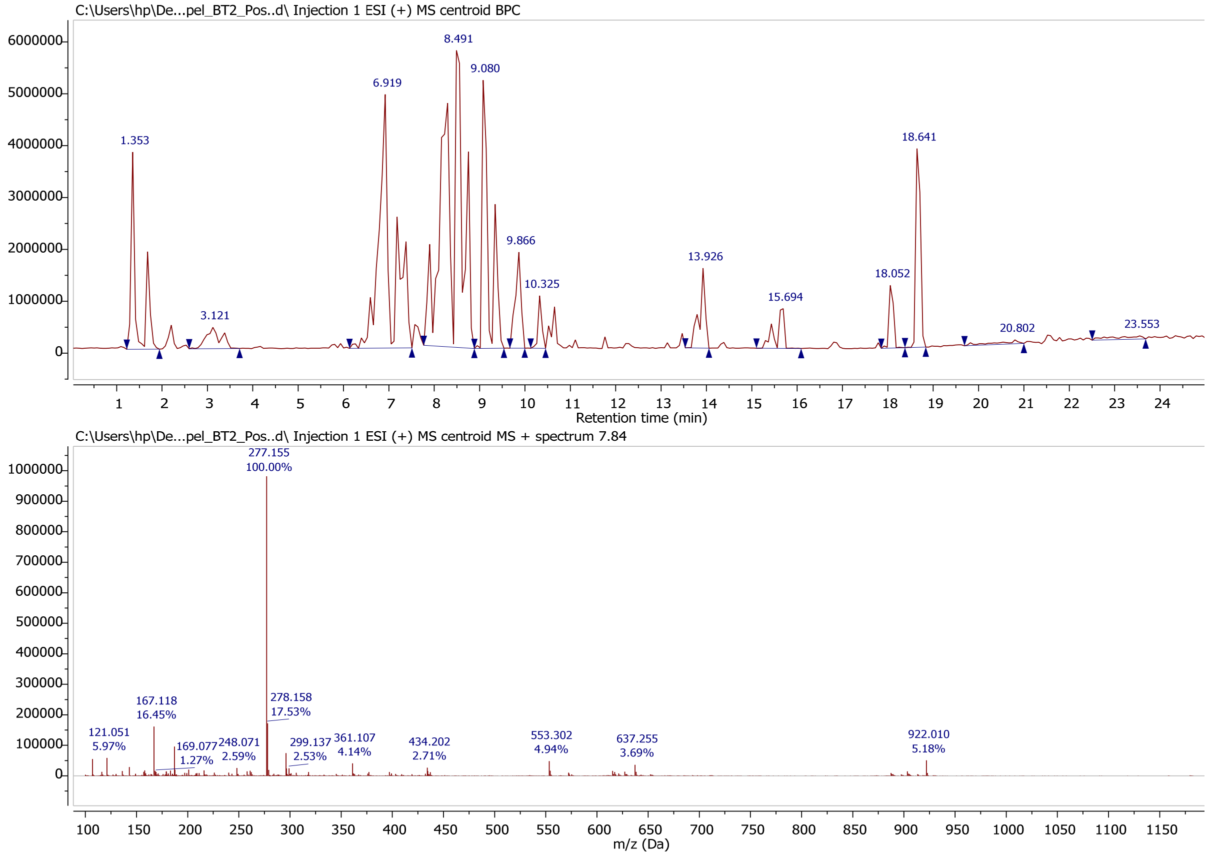

Figure S25. BPC and MS profile of cyclo-(Tyr-Leu) (**21**)

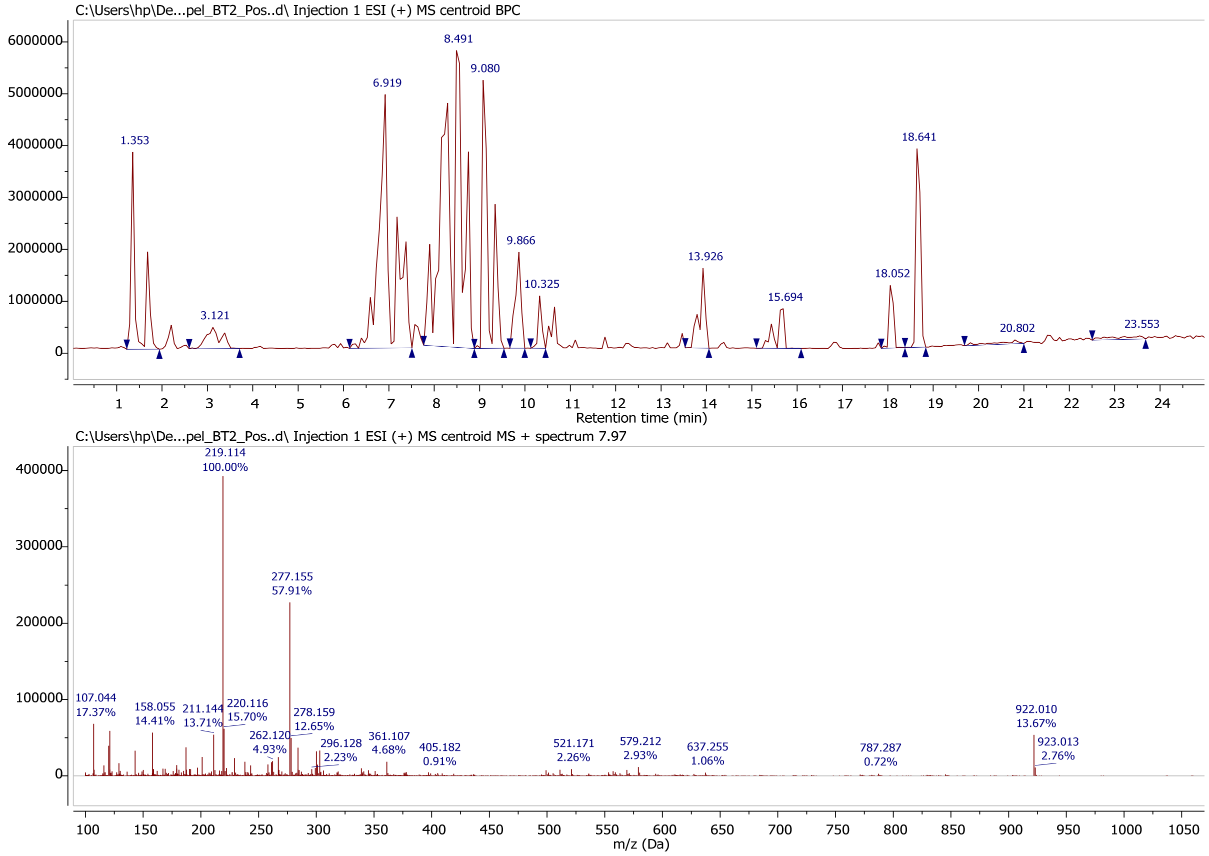

Figure S26. BPC and MS profile of cyclo-(L-Phe-L-Ala) (**22**)

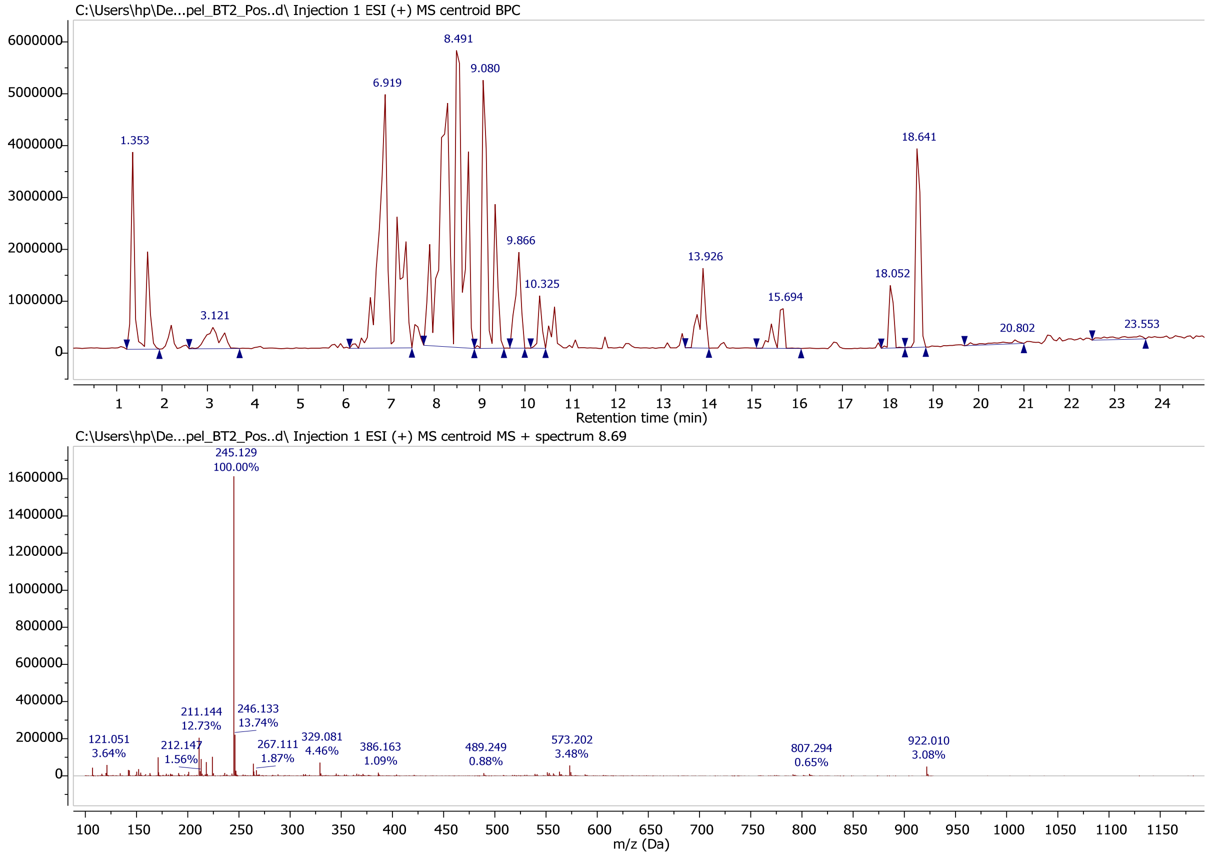

Figure S27. BPC and MS profile of cyclo-(phenylalanyl-prolyl) (**23**)

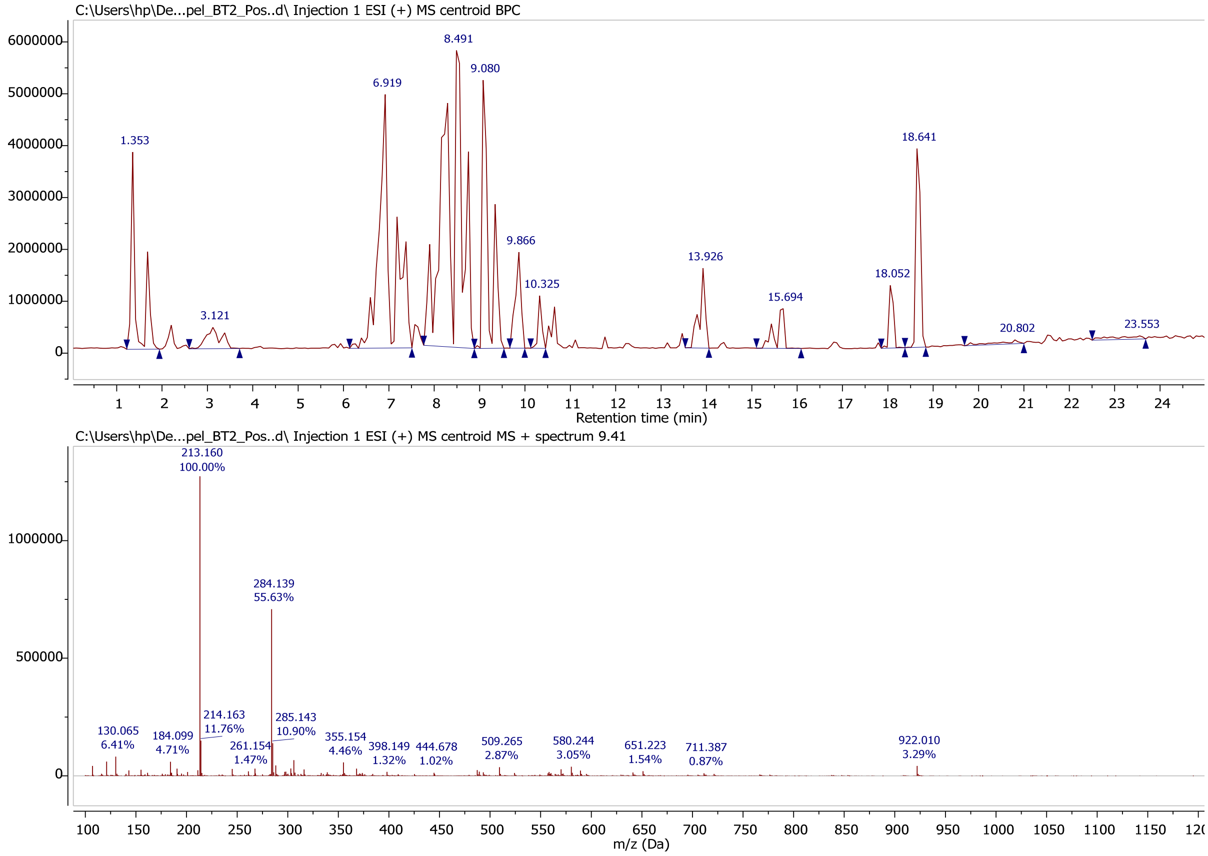

Figure S28. BPC and MS profile of brevianamide F (**24**)

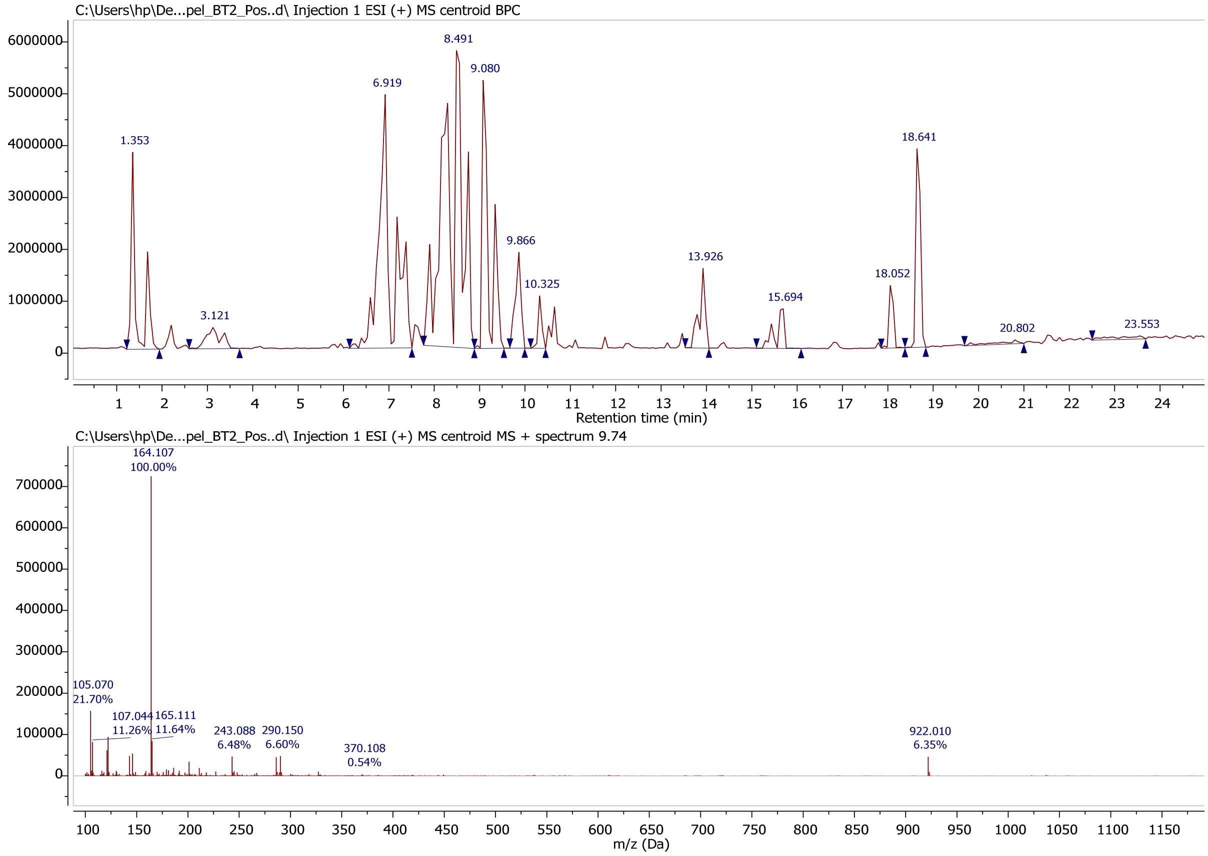

Figure S29. BPC and MS profile of N-phenethylacetamide (**25**)

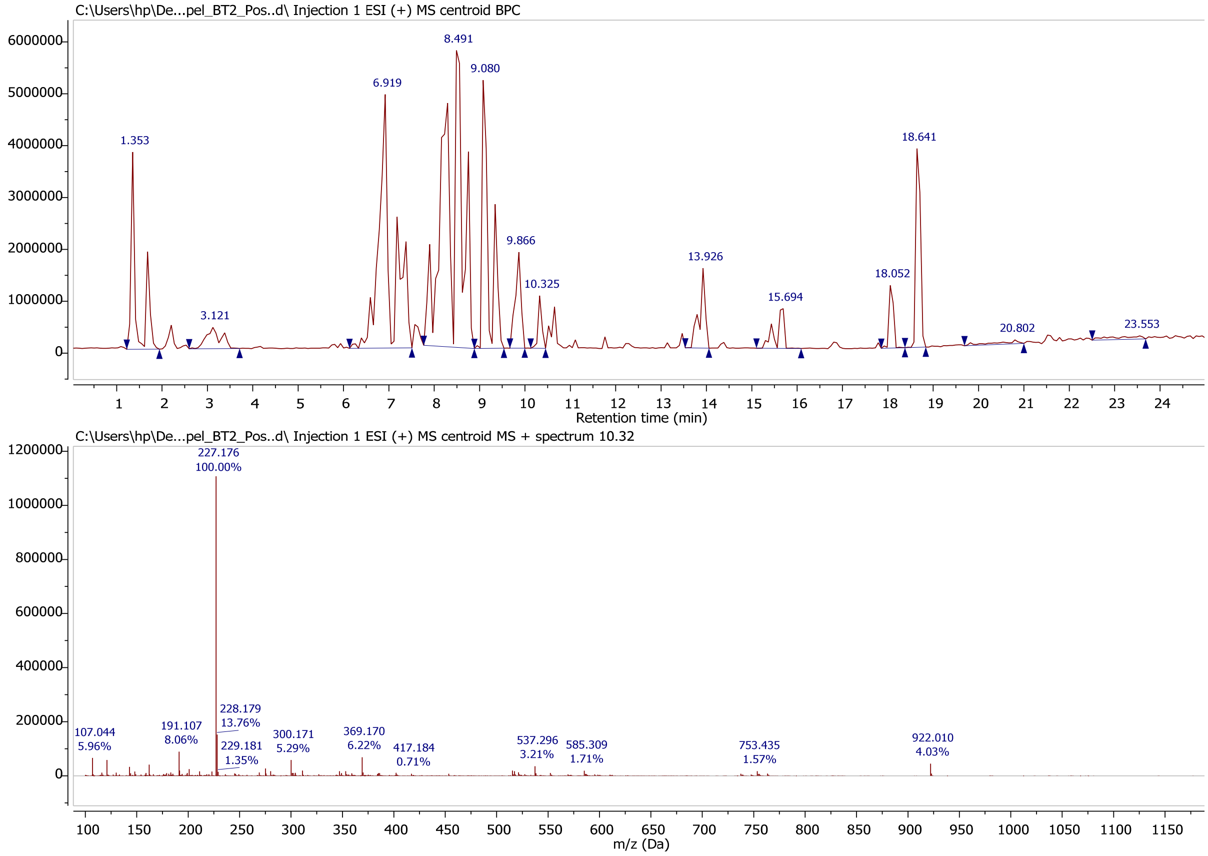

Figure S30. BPC and MS profile of cyclo-(L-leucyl-L-leucyl) (**26**)

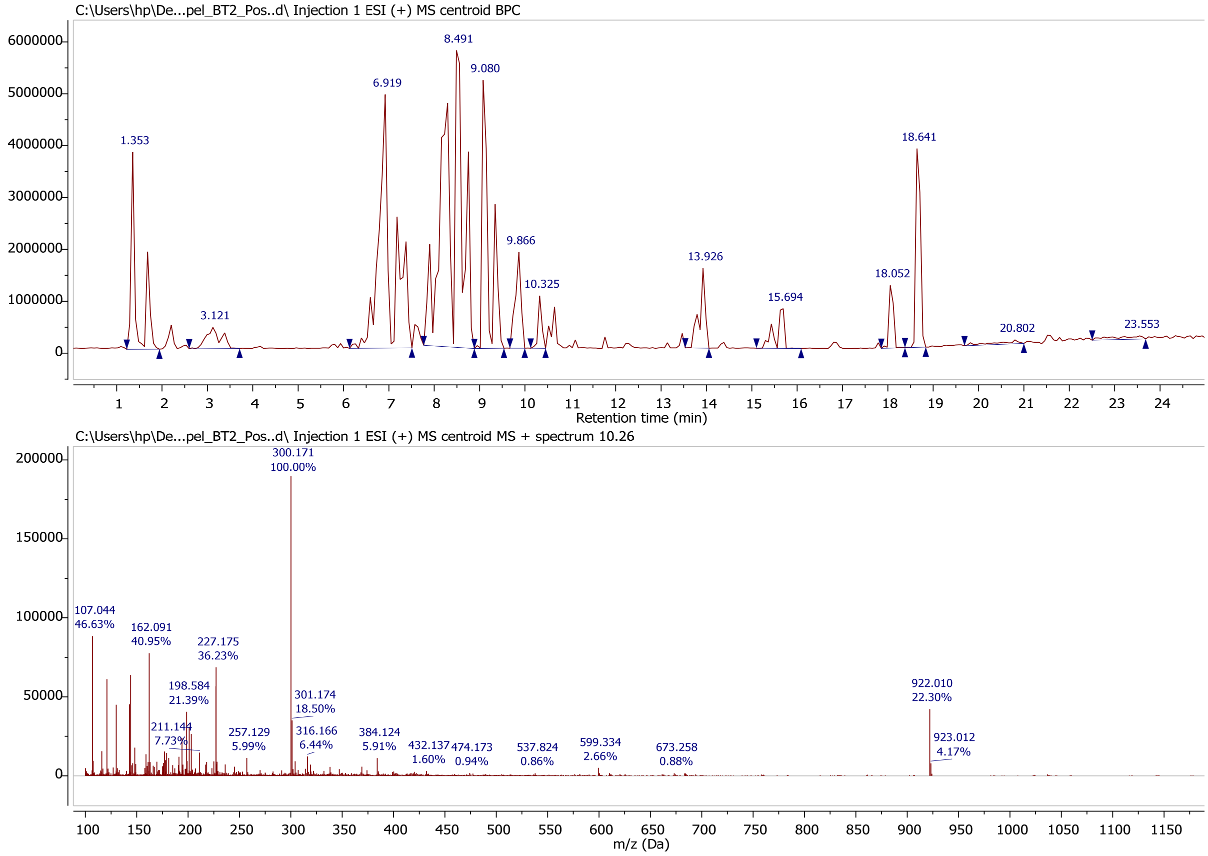

Figure S31. BPC and MS profile of cyclo-(D-Leu-L-Trp) (**27**)

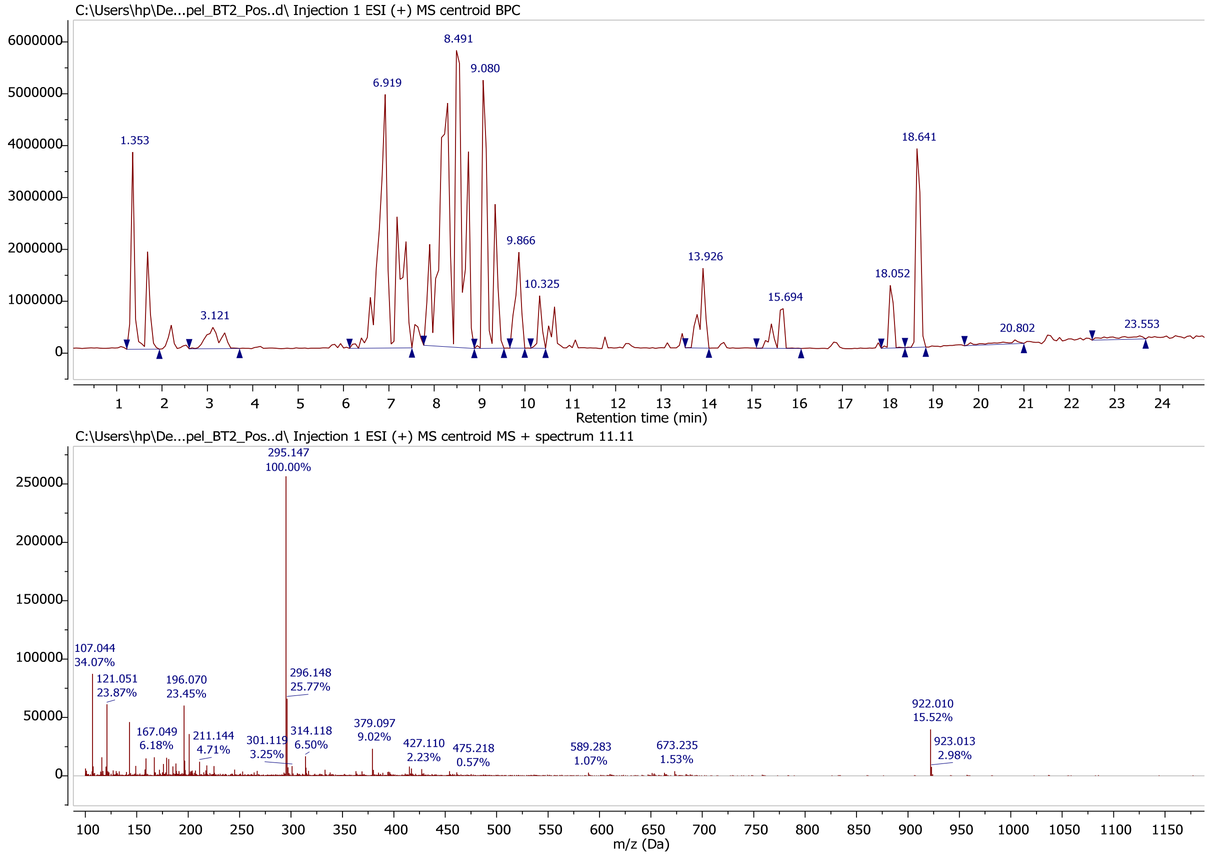

Figure S32. BPC and MS profile of cyclo-(phenylalanyl-phenylalanyl) (**28**)

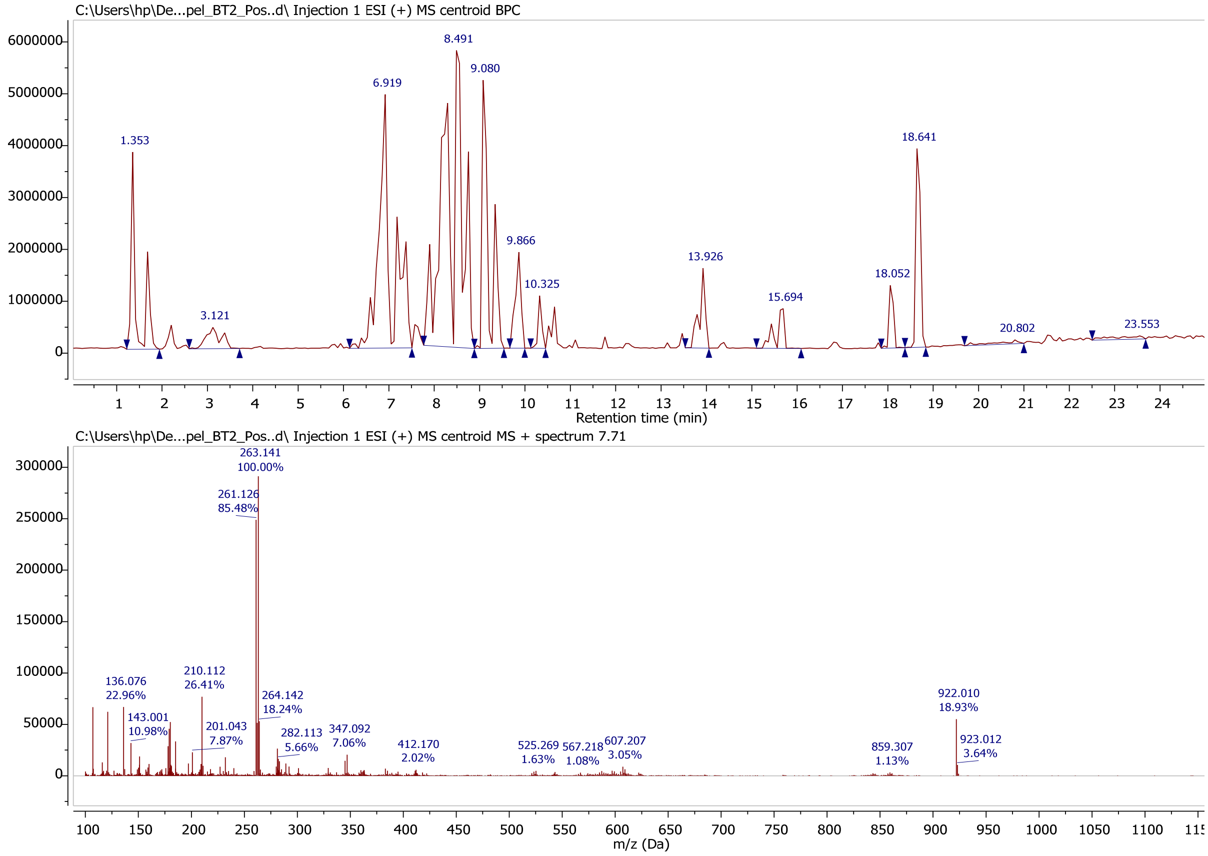

Figure S33. BPC and MS profile of cyclo-(Tyr-Val) (**29**)

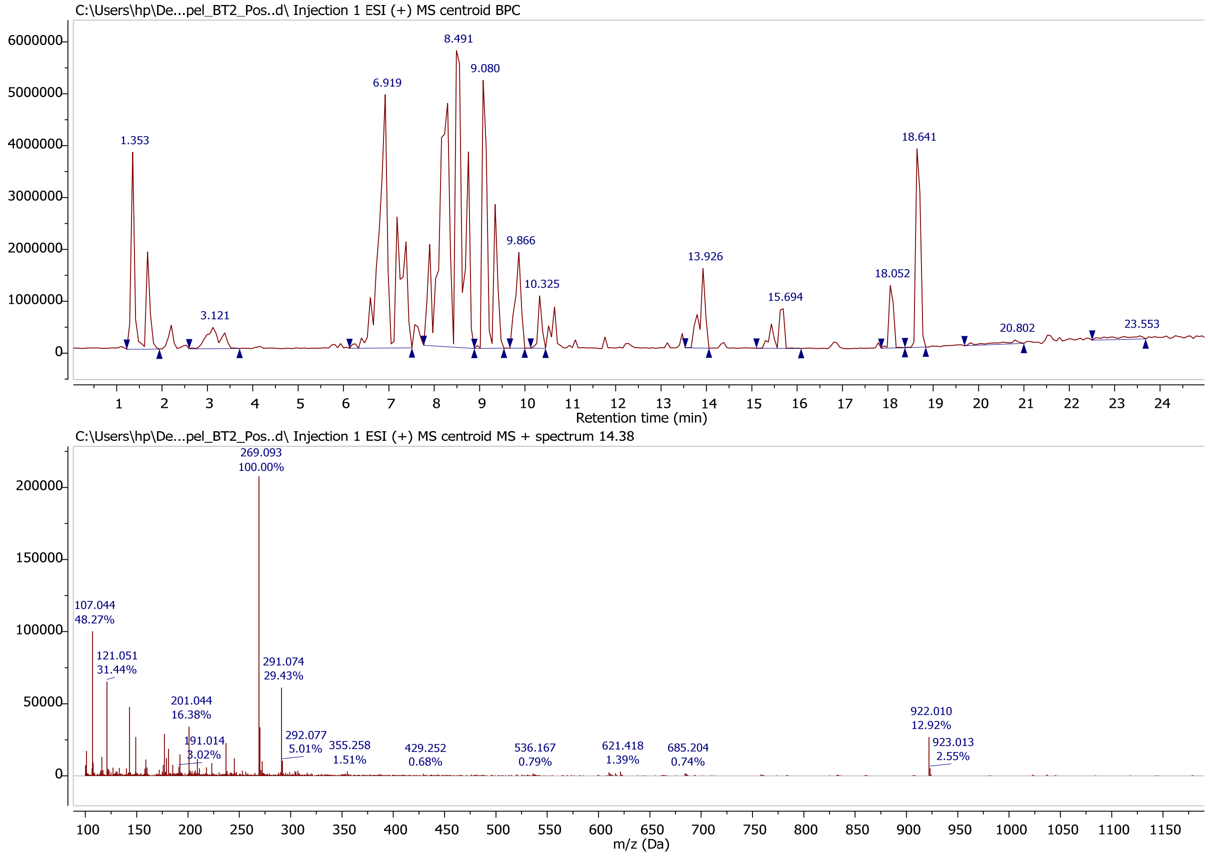

Figure S34. BPC and MS profile of 1-acetyl-3-methoxycarbonyl-β-carboline (**30**)

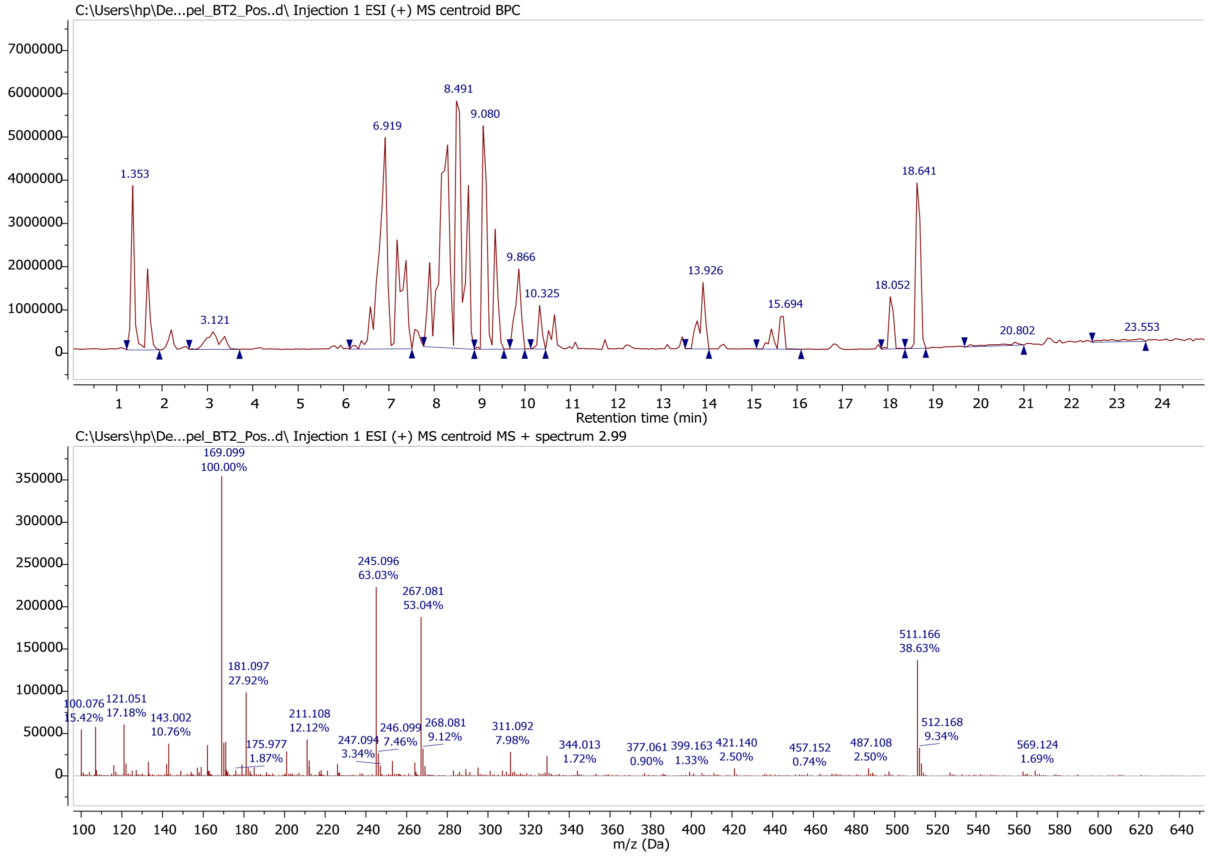

Figure S35. BPC and MS profile of cyclo-(D-Ala-L-Pro) (**31**)

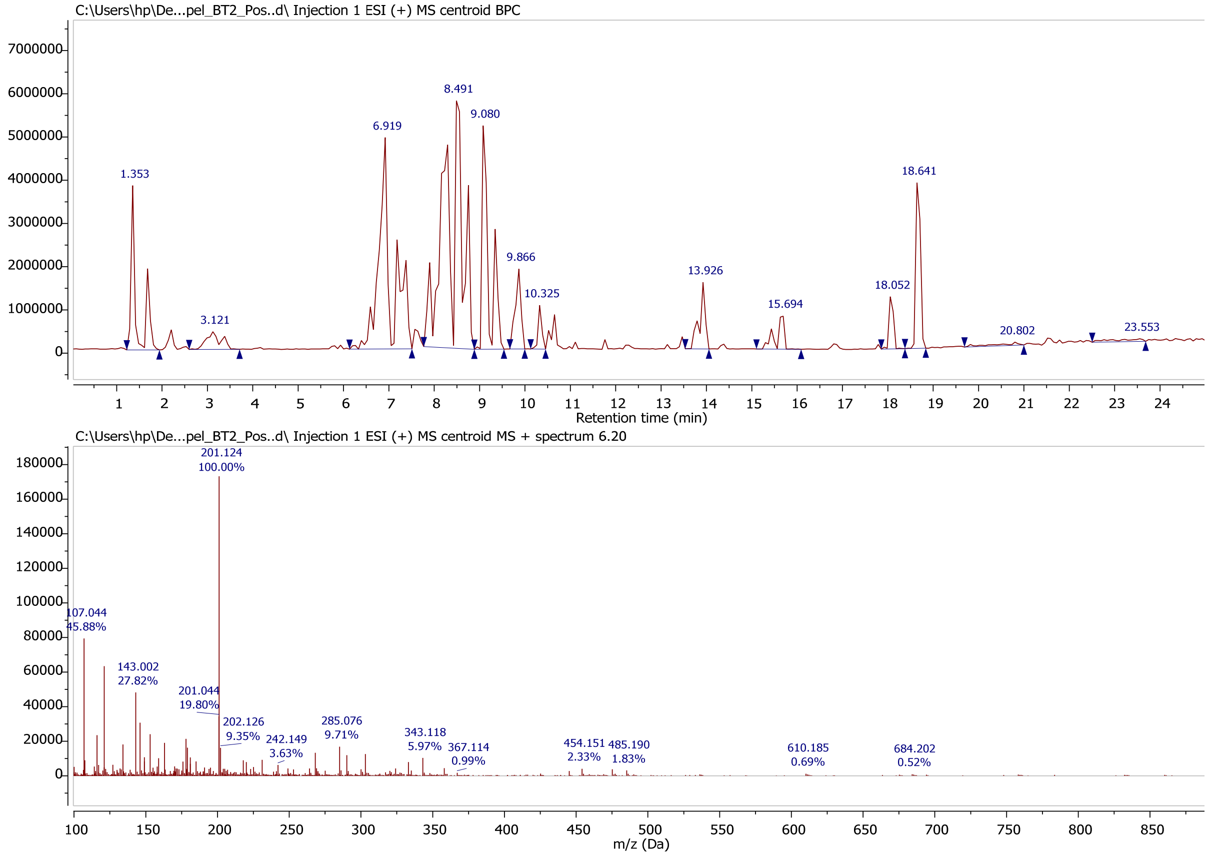

Figure S36. BPC and MS profile of cyclo-(Ile-Ser) (**32**)

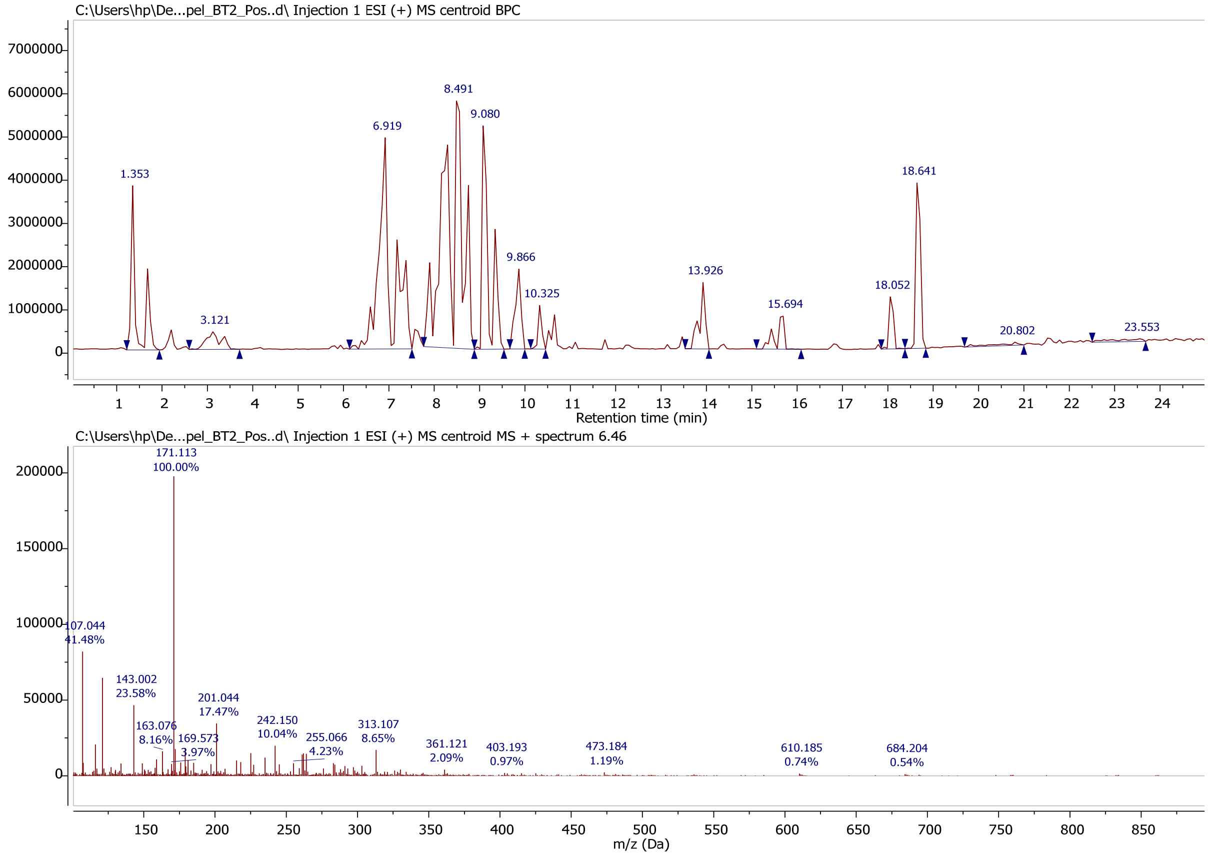

Figure S37. BPC and MS profile of (S)-3-isobutylpiperazine-2,5-dione (**33**)

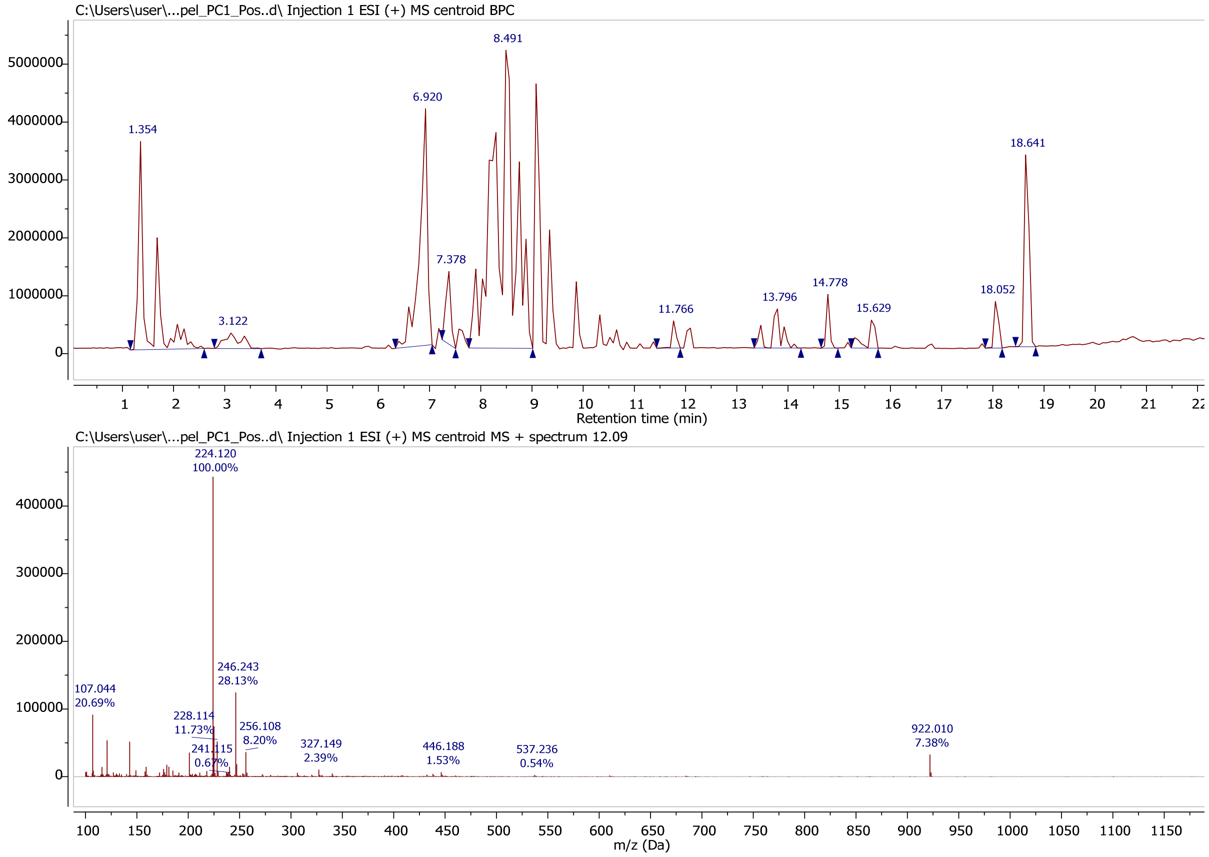

Figure S38. BPC and MS profile of 3-[(6-methylpyrazin-2-yl) methyl]-1H-indole (**34**)

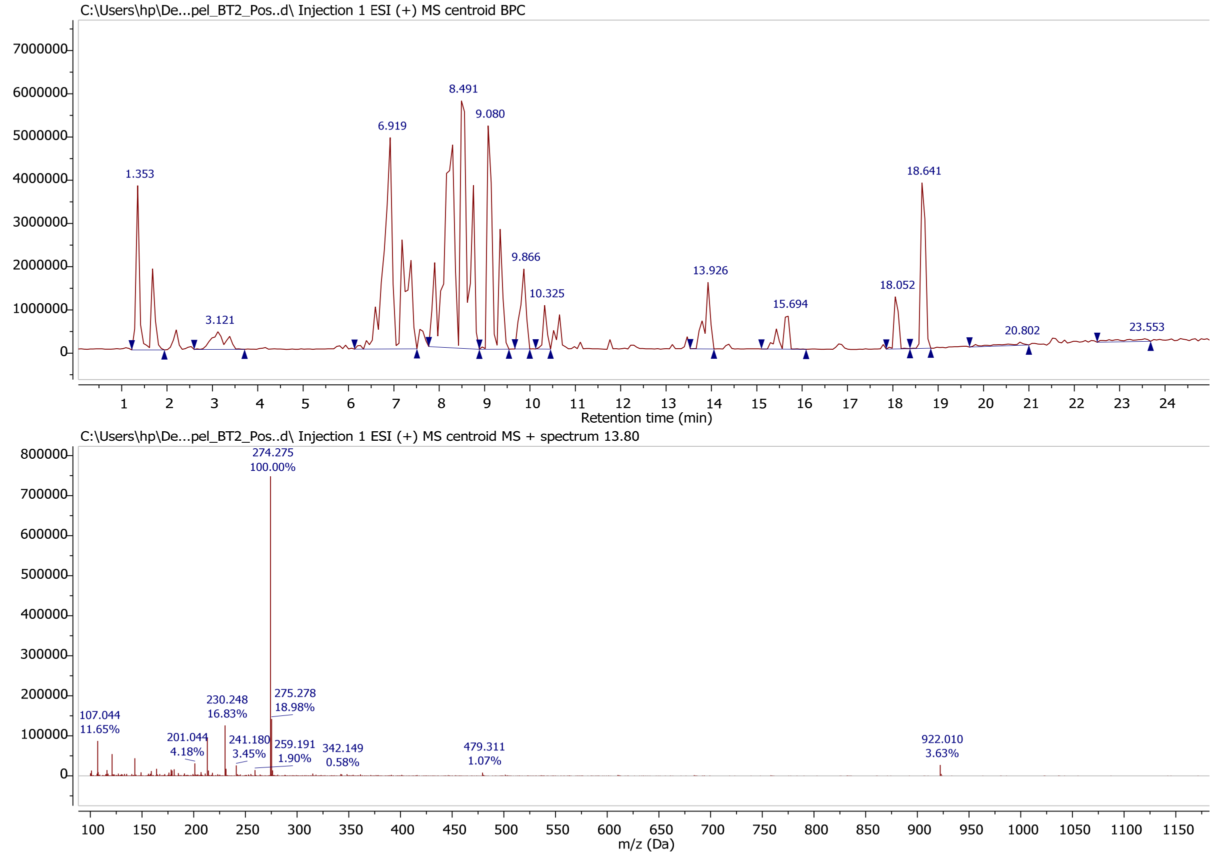

Figure S39. BPC and MS profile of N-lauryldiethanolamine (**35**)

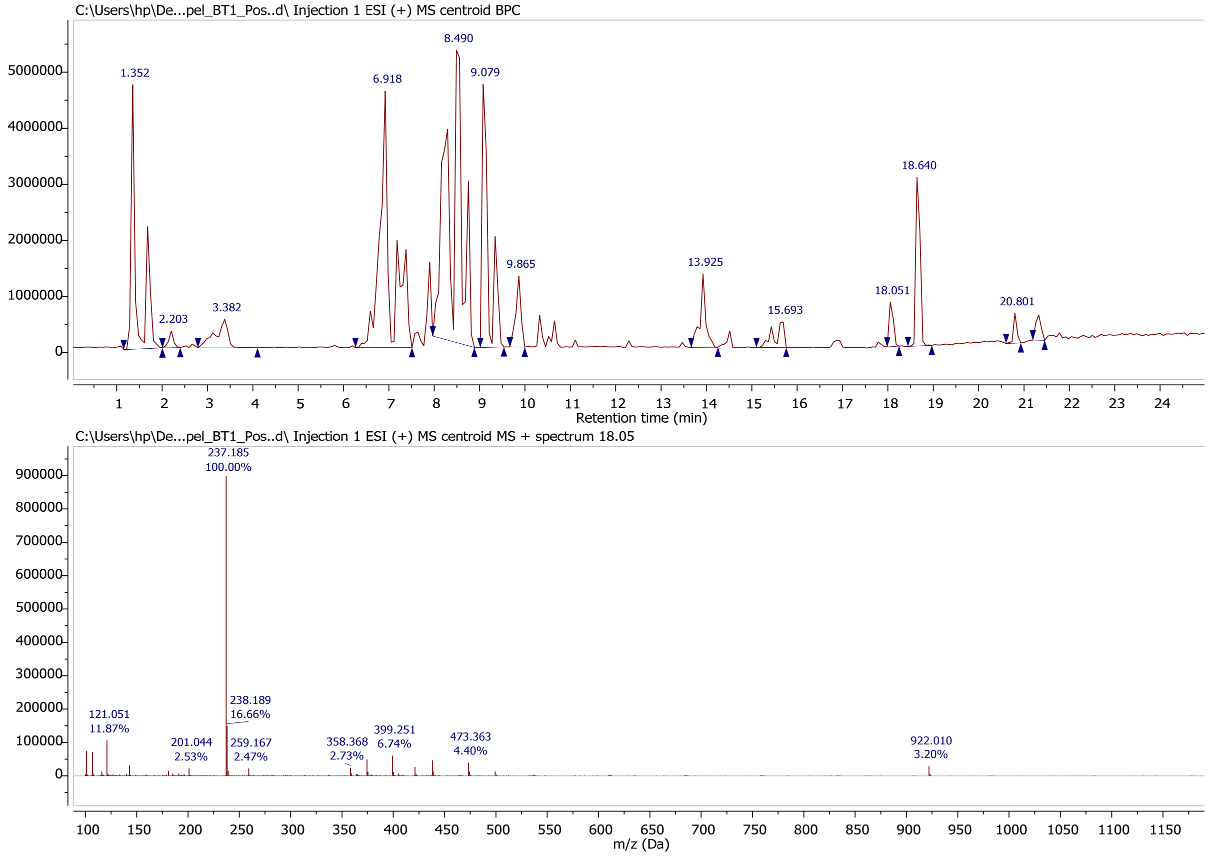

Figure S40. BPC and MS profile of 2-n-hexyl-5-n-propylresorcinol (**36**)

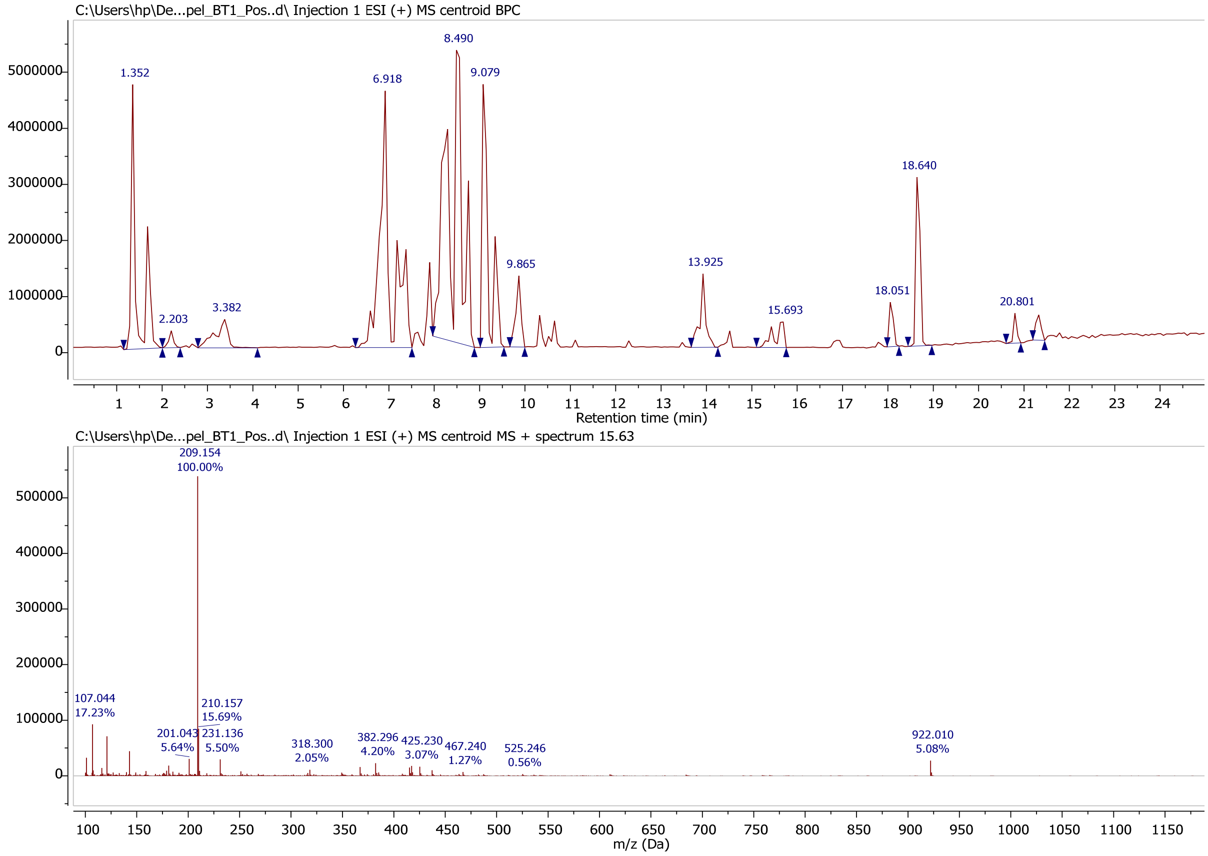

Figure S41. BPC and MS profile of 2-hexyl-5-methyl resorcinol (**37**)
